# Supplementary material for: The impact of liquefaction disaster on farming systems at agriculture land based on technical and psychosocial perspectives
Source: PLoS One. 2021 Jan 25;16(1):e0245591. doi: 10.1371/journal.pone.0245591 (PMC7834136; doi:10.1371/journal.pone.0245591)
Supplement: S1 Data — (ZIP) [file pone.0245591.s001.zip › LINK Research Map.docx]

<https://www.google.com/maps/d/edit?mid=14lZetnL2icqv2MPRUgppDIF-MejsLCAT&usp=sharing>
