# Supplementary material for: The impact of liquefaction disaster on farming systems at agriculture land based on technical and psychosocial perspectives
Source: PLoS One. 2021 Jan 25;16(1):e0245591. doi: 10.1371/journal.pone.0245591 (PMC7834136; doi:10.1371/journal.pone.0245591)

Peta Jono Oge

Lapisan tanpa judul

- Jono Oge Regency
- Jono Oge Regency
- Petobo Regency
- Petobo

Peta Gempa

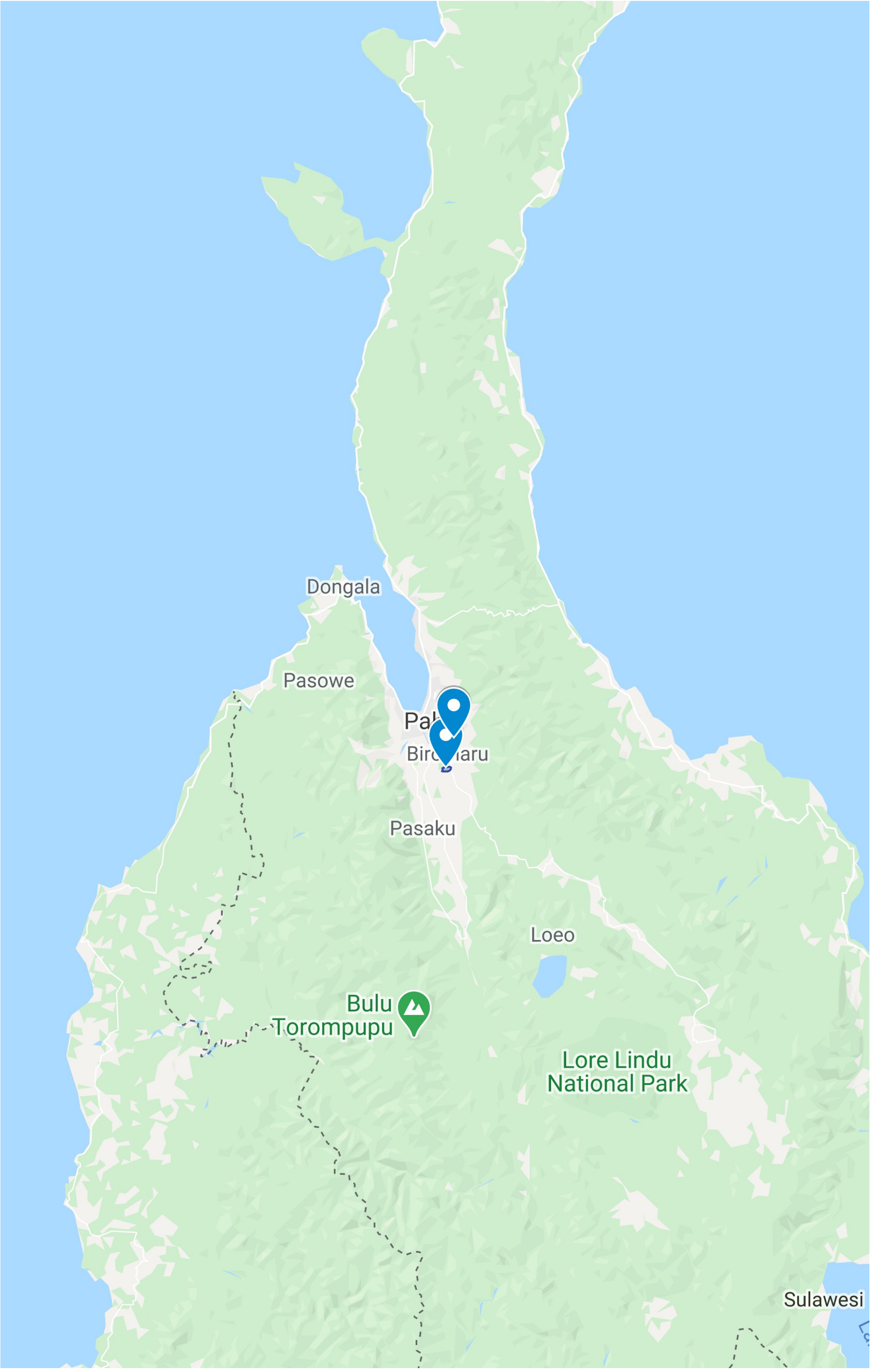

Supplement: S1 Data — (ZIP) [file pone.0245591.s001.zip › Research Map.pdf]
