# Supplementary material for: The impact of liquefaction disaster on farming systems at agriculture land based on technical and psychosocial perspectives
Source: PLoS One. 2021 Jan 25;16(1):e0245591. doi: 10.1371/journal.pone.0245591 (PMC7834136; doi:10.1371/journal.pone.0245591)
Supplement: S1 Data — (ZIP) [file pone.0245591.s001.zip › Support Data Table 3 dan Table 4.docx]

**Uptake of Sweet Corn (Zea Mays Saccarata Sturth) Phosphorus due to Chicken Coop Fertilizer and SP-36 Fertilizer on Post-Liquefaction of Sidera**

The factorial randomized block design (RBD) consisted of 2 factors. The first factor (chicken manure) consists of 4 levels: P0 (0 tonnes ha^-1^ or without chicken manure 0 kg plot -1), P1 (10 tons / ha of chicken manure equivalent to 5 kg plot-1), P2 (25 tons ha-1 chicken manure equivalent to 12.5 kg plot-1) and P3 40 tons ha- 1 chicken manure is equivalent to 20 kg plot-1) while the second factor (Fertilizer SP-36) consists of 4 levels: S0 (0 kg ha^-1^ or without SP-36 fertilizer), S1 (100 kg ha-1 or 50 g P plot-1), S2 (200 kg ha-1 or 100 g P plot-1) and S3 (300 kg ha-1 or 150 g Plot-1).

The results showed that the dose of chicken manure40 tonnes ha-1and fertilizer SP-36 at a dose of 300 kg ha-1, it has been shown to significantly increase soil H2O pH, total P, available P, plant dry weight, phosphorus absorption and ear weight without husks.

**Initial Soil Analysis**

| **No.** | **Parameter** | **Unit** | **Score** | **Information** |
| --- | --- | --- | --- | --- |
| 1 | Sand | % | 60.9 |  |
| 2 | Dust | % | 10.2 | Clay sand  argillaceous |
| 3 | Clay | % | 28.9 |  |
| 5 | Soil weight | g / cm3 | 1.6 |  |
| 7 | C-organic | % | 1.02 | Low |
| 8 | N-Total | % | 0.16 | Low |
| 9 | C / N |  | 8.5 | Low |
| 10 | pH H2O (1: 2.5) | | 5.8 | It's a little sour |
| 11 | pH KCl (1: 2.5) | | 4,9 |  |
| 12 | P2O5 (HCl 25%) | mg / 100g | 24.08 | Low |
| 13 | P2O5 (Bray I) | Ppm | 10.33 | Low |
| 14 | K2O (HCl 25%) | me / 100g | 29.41 | Moderate |
| 15 | Ca | me / 100g | 4.65 | Low |
| 16 | Mg | me / 100g | 0.43 | Low |
| 17 | K | me / 100g | 0.25 | Low |
| 18 | Na | me / 100g | 0.17 | Low |
| 19 | KTK | me / 100g | 23.27 | Moderate |
| 20 | KB | % | 23.63 | Low |
| 21 | Al-dd | me / 100g | 0.35 |  |
| 22 | H-dd | me / 100g | 0.3 |  |

**Chicken Cage Fertilizer Analysis**

| No. | Sample code | Nitrogen | Phosphorous | Potassium | C-organic | C / N ratio |
| --- | --- | --- | --- | --- | --- | --- |
|  |  | (%) | (%) | (%) | (%) | Ratio |
| 1 | Chicken manure | 1.95 | 0.1 | 0.2 | 21.56 | 11.10 |

**Soil Chemical Properties Assessment Criteria**

| **Nature of Soil** | **Very** | **Low** | **Moderate** | **High** | **Very** |
| --- | --- | --- | --- | --- | --- |
|  | **Low** |  |  |  | **high** |
| C (%) | <1.00 | 1.00-2.00 | 2.01-3.00 | 3.01-5.00 | > 5.00 |
| N (%) | <0.10 | 0.10-0.20 | 0.21-0.50 | 0.51-0.75 | > 0.75 |
| C / N | <5 | 5-10 | 11-15 | 16-25 | > 25 |
| P2O5 HCl 25%  (mg / 100 g) | <10 | 21-40 | 21-40 | 41-60 | > 60 |
| P2O5 Bray (ppm) | <10 | 10-15 | 16-25 | 26-35 | > 35 |
| P2O5 Olsen (ppm) | <10 | 10-25 | 26-45 | 46-60 | > 60 |
| K2O HCl 25% (mg / 100 g) | <10 | 10-20 | 21-40 | 41-60 | > 60 |
| CEC (mg / 100 g) | <5 | 5-16 | 17-24 | 25-40 | > 40 |
| **Arrangement of Cations** |  |  |  |  |  |
| K (me / 100 g) | <0.1 | 0.1-0.2 | 0.3-0.5 | 0.6-1.0 | > 1.0 |
| Na (me / 100 g) | <0.1 | 0.1-0.2 | 0.4-0.7 | 0.8-1.0 | > 1.0 |
| Mg (me / 100 g) | <0.4 | 0.4-1.0 | 1.1-2.0 | 2,1-8,0 | > 8.0 |
| Ca (me / 100 g) | <0.2 | 2-5 | 6-10 | 11-20 | > 20 |
| Base Saturation (%) | <20 | 20-35 | 36-50 | 51-70 | > 70 |
| Al Saturation (%) | <10 | 10-20 | 21-30 | 31-60 | > 60 |
| **Very** | **Sour** | **Kinda** | **Neutral** | **Kinda** | **Alkalis** |
| **Sour** |  | **Sour** |  | **Alkalis** |  |
| pH H2O <4.5 | 4.5-5.5 | 5,6-6,5 | 6,6-7,5 | 7,6-8,5 | **>**8.5 |

**Observation of soil pH (H2O)**

| Combination Treatment | | Deuteronomy | | | amount | Average |
| --- | --- | --- | --- | --- | --- | --- |
| Chicken Manure (A) | SP-36 (B) | 1 | 2 | 3 |  |  |
| P0 | S0 | 5.33 | 5.36 | 5.32 | 16.01 | 5.34 |
|  | S1 | 5.48 | 5.49 | 5.47 | 16.44 | 5.48 |
|  | S2 | 5.62 | 5.66 | 5.64 | 16.92 | 5.64 |
|  | S3 | 5.78 | 5.79 | 5.8 | 17.37 | 5.79 |
| P1 | S0 | 5,57 | 5,56 | 5,58 | 16.71 | 5,57 |
|  | S1 | 5.78 | 5.77 | 5.76 | 17.31 | 5.77 |
|  | S2 | 5.83 | 5.86 | 5.84 | 17.53 | 5.84 |
|  | S3 | 5.93 | 5.94 | 5.95 | 17.82 | 5.94 |
| P2 | S0 | 5.89 | 5.85 | 5.88 | 17.62 | 5.87 |
|  | S1 | 6.03 | 6,08 | 6.04 | 18.15 | 6.05 |
|  | S2 | 6.18 | 6.19 | 6.2 | 18.57 | 6.19 |
|  | S3 | 6.28 | 6.27 | 6.29 | 18.84 | 6.28 |
| P3 | S0 | 5.91 | 5.89 | 5,9 | 17.7 | 5.90 |
|  | S1 | 6.18 | 6.2 | 6.17 | 18.55 | 6.18 |
|  | S2 | 6.25 | 6.23 | 6.24 | 18.72 | 6.24 |
|  | S3 | 6.39 | 6.37 | 6.38 | 19.14 | 6.38 |
| amount |  | 94.43 | 94.51 | 94.46 | 283.4 | 5.90 |

**Soil pH (H2O) Variety Scanning Table**

| Source of Diversity | Level | amount | Square | F | F Table | |
| --- | --- | --- | --- | --- | --- | --- |
|  | Free | Square | Middle | Count | 1% | 5% |
| Group | 2 | 0.000204 | 0.000102083 | 0.490306 | 5.39 | 3.32 |
| AxB combination | 15 | 4,1453 | 0.276353333 | 1327,326 ** | 2.7 | 2.01 |
| Chicken Fertilizer | 3 | 2,92825 | 0.976083333 | 4688.13 ** | 4.51 | 2.92 |
| SP - 36 | 3 | 1,185883 | 0.395294444 | 1898.6 ** | 4.51 | 2.92 |
| Interaction | 9 | 0.031167 | 0.003462963 | 16,63262 ** | 3.07 | 2.21 |
| Error | 32 | 0.006662 | 0.000208203 |  |  |  |
| **Total** | 47 | 4.152167 | KK | | 0.594 | |

Note: ** = Very Real Impact

**Observation P-Total Soil (mg / 100 g)**

| Combination Treatment | | Deuteronomy | | | amount | Average |
| --- | --- | --- | --- | --- | --- | --- |
| Chicken Manure (A) | SP-36 (B) | 1 | 2 | 3 |  |  |
| P0 | S0 | 24.08 | 24.06 | 24.07 | 72.21 | 24.07 |
|  | S1 | 27.02 | 27.04 | 27.03 | 81.09 | 27.03 |
|  | S2 | 29.67 | 29.68 | 29.69 | 89.04 | 29.68 |
|  | S3 | 32.12 | 32.11 | 32.13 | 96.36 | 32.12 |
| P1 | S0 | 28.45 | 28.43 | 28.44 | 85.32 | 28.44 |
|  | S1 | 31.89 | 31.9 | 31.91 | 95.7 | 31.90 |
|  | S2 | 33.61 | 33.62 | 3, .6 | 100.83 | 33.61 |
|  | S3 | 35.47 | 35.45 | 35.46 | 106.38 | 35.46 |
| P2 | S0 | 32.17 | 32.18 | 32.19 | 96.54 | 32.18 |
|  | S1 | 35.07 | 35.08 | 35.09 | 105.24 | 35.08 |
|  | S2 | 37.72 | 37.7 | 37.71 | 113.13 | 37.71 |
|  | S3 | 40.83 | 40.85 | 40.84 | 122.52 | 40.84 |
| P3 | S0 | 35.82 | 35.84 | 35.83 | 107.49 | 35.83 |
|  | S1 | 38.53 | 38.52 | 38.51 | 115.56 | 38.52 |
|  | S2 | 44.65 | 44,67 | 44,66 | 133,98 | 44,66 |
|  | S3 | 52,54 | 52,53 | 52,52 | 157,59 | 52,53 |
| Total |  | 559,64 | 559,66 | 559,68 | 1678,98 | 34,98 |

**Check Table of P-Total Land Variety**

| Source of Diversity | Level | amount | Square | F | F Table | |
| --- | --- | --- | --- | --- | --- | --- |
|  | Free | Square | Middle | Count | 1% | 5% |
| Group | 2 | 5E-05 | 2.5E-05 | 0.253968 | 5.39 | 3.32 |
| AxB combination | 15 | 2212,401 | 147,4934 | 1498346 ** | 2.7 | 2.01 |
| Chicken Fertilizer | 3 | 1406,293 | 468,7642 | 4762049 ** | 4.51 | 2.92 |
| SP - 36 | 3 | 679,6354 | 226,5451 | 2301411 ** | 4.51 | 2.92 |
| Interaction | 9 | 126,4729 | 14.05254 | 142756 ** | 3.07 | 2.21 |
| Error | 32 | 0.00315 | 9.84E-05 |  |  |  |
| Total | 47 | 2212,404 | KK | | 0.168 | |

Note: ** = Very Real Impact

**Observation of P-Available Soil (ppm)**

| Combination Treatment | | Deuteronomy | | | amount | Average |
| --- | --- | --- | --- | --- | --- | --- |
| Chicken Manure (A) | SP-36 (B) | 1 | 2 | 3 |  |  |
| P0 | S0 | 10.11 | 10,12 | 10.14 | 30.37 | 10,12 |
|  | S1 | 12.52 | 12.56 | 12.54 | 37.62 | 12.54 |
|  | S2 | 14.36 | 14.37 | 14.35 | 43.08 | 14.36 |
|  | S3 | 15.24 | 15.23 | 15.22 | 45.69 | 15.23 |
| P1 | S0 | 12.34 | 12.36 | 12.35 | 37.05 | 12.35 |
|  | S1 | 14.85 | 14.87 | 14.89 | 44.61 | 14.87 |
|  | S2 | 16.34 | 16.33 | 16.35 | 49.02 | 16.34 |
|  | S3 | 17.21 | 17.2 | 17.22 | 51.63 | 17.21 |
| P2 | S0 | 15.12 | 15.13 | 15.14 | 45.39 | 15.13 |
|  | S1 | 16.77 | 16.78 | 16.76 | 50.31 | 16.77 |
|  | S2 | 19.8 | 19.82 | 19.81 | 59.43 | 19.81 |
|  | S3 | 21.24 | 21.23 | 21.25 | 63.72 | 21.24 |
| P3 | S0 | 17.92 | 17.91 | 17.93 | 53.76 | 17.92 |
|  | S1 | 23.86 | 23.85 | 23.87 | 71.58 | 23.86 |
|  | S2 | 26.23 | 26.22 | 26.24 | 78.69 | 26.23 |
|  | S3 | 28.88 | 28.86 | 28.87 | 86.61 | 28.87 |
| amount |  | 282.79 | 282.84 | 282.93 | 848.56 | 17.68 |

**Check Table of Variety P-Available Land**

| Source of Diversity | Level | amount | Square | F | F Table | |
| --- | --- | --- | --- | --- | --- | --- |
|  | Free | Square | Middle | Count | 1% | 5% |
| Group | 2 | 0.000629 | 0.000315 | 2.493292 | 5.39 | 3.32 |
| AxB combination | 15 | 1199,937 | 79.99577 | 634022.2 ** | 2.7 | 2.01 |
| Chicken Fertilizer | 3 | 847,0039 | 282,3346 | 2237699 ** | 4.51 | 2.92 |
| SP - 36 | 3 | 310,7326 | 103,5775 | 820924.2 ** | 4.51 | 2.92 |
| Interaction | 9 | 42.2 | 4.688889 | 37162.71 ** | 3.07 | 2.21 |
| Error | 32 | 0.004038 | 0.000126 |  |  |  |
| Total | 47 | 1199,941 | KK | | 0.267 | |

Note: ** = Very Real Impact

**. Observation of Plant Dry Weight (g plot-1)**

| Combination Treatment | | Deuteronomy | | | amount | Average |
| --- | --- | --- | --- | --- | --- | --- |
| Chicken Manure (A) | SP-36 (B) | 1 | 2 | 3 |  |  |
| P0 | S0 | 275 | 301 | 279 | 855 | 285.00 |
|  | S1 | 318 | 321 | 315 | 954 | 318.00 |
|  | S2 | 354 | 351 | 356 | 1061 | 353.67 |
|  | S3 | 466 | 468 | 461 | 1395 | 465.00 |
| P1 | S0 | 352 | 355 | 357 | 1064 | 354.67 |
|  | S1 | 379 | 376 | 377 | 1132 | 377.33 |
|  | S2 | 391 | 394 | 395 | 1180 | 393.33 |
|  | S3 | 566 | 561 | 563 | 1690 | 563.33 |
| P2 | S0 | 448 | 446 | 443 | 1337 | 445.67 |
|  | S1 | 532 | 536 | 535 | 1603 | 534.33 |
|  | S2 | 585 | 583 | 581 | 1749 | 583.00 |
|  | S3 | 608 | 610 | 609 | 1827 | 609.00 |
| P3 | S0 | 477 | 475 | 478 | 1430 | 476.67 |
|  | S1 | 562 | 564 | 566 | 1692 | 564.00 |
|  | S2 | 667 | 663 | 665 | 1995 | 665.00 |
|  | S3 | 711 | 713 | 718 | 2142 | 714.00 |
| amount |  | 7691 | 7717 | 7698 | 23106 | 481.38 |

**Scanning Table of Variety of Plant Dry Weight**

| Source of Diversity | Level | amount | Square | F | F Table | |
| --- | --- | --- | --- | --- | --- | --- |
|  | Free | Square | Middle | Count | 1% | 5% |
| Group | 2 | 22,625 | 11,3125 | 0.667025 | 5.39 | 3.32 |
| AxB combination | 15 | 745671.9 | 49711.46 | 2931,163 ** | 2.7 | 2.01 |
| Chicken Fertilizer | 3 | 461175.8 | 153725.3 | 9064,184 ** | 4.51 | 2.92 |
| SP - 36 | 3 | 251757.4 | 83919.14 | 4948,169 ** | 4.51 | 2.92 |
| Interaction | 9 | 32738.75 | 3637,639 | 214,488 ** | 3.07 | 2.21 |
| Error | 32 | 542,7083 | 16,95964 |  |  |  |
| Total | 47 | 746237.3 | KK | | 18,770 | |

Note: ** = Very Real Impact

**Observation of Plant P Uptake (g plant-1)**

| Combination Treatment | | Deuteronomy | | | amount | Average |
| --- | --- | --- | --- | --- | --- | --- |
| Chicken Manure (A) | SP-36 (B) | 1 | 2 | 3 |  |  |
| P0 | S0 | 0.14 | 0.15 | 0.13 | 0.42 | 0.140 |
|  | S1 | 0.207 | 0.196 | 0.217 | 0.62 | 0.207 |
|  | S2 | 0.266 | 0.232 | 0.296 | 0.94 | 0.265 |
|  | S3 | 0.363 | 0.317 | 0.332 | 1,012 | 0.337 |
| P1 | S0 | 0.225 | 0.238 | 0.217 | 0.68 | 0.227 |
|  | S1 | 0.269 | 0.288 | 0.279 | 0.836 | 0.279 |
|  | S2 | 0.305 | 0.375 | 0.355 | 1,035 | 0.345 |
|  | S3 | 0.475 | 0.434 | 0.406 | 1,315 | 0.438 |
| P2 | S0 | 0.327 | 0.314 | 0.297 | 0.938 | 0.313 |
|  | S1 | 0.397 | 0.409 | 0.426 | 1,232 | 0.411 |
|  | S2 | 0.469 | 0.482 | 0.484 | 1,435 | 0.478 |
|  | S3 | 0.598 | 0.549 | 0.535 | 1,682 | 0.561 |
| P3 | S0 | 0.372 | 0.389 | 0.402 | 1,163 | 0.388 |
|  | S1 | 0.489 | 0.498 | 0.505 | 1,492 | 0.497 |
|  | S2 | 0.607 | 0.608 | 0.595 | 1.81 | 0.603 |
|  | S3 | 0.697 | 0.712 | 0.641 | 2.05 | 0.683 |
| amount |  | 6,206 | 6,191 | 6,117 | 18,514 | 0.386 |

**Scanning Table of Plant P Uptake Variety**

| Source of Diversity | Level | amount | Square | F | F Table | |
| --- | --- | --- | --- | --- | --- | --- |
|  | Free | Square | Middle | Count | 1% | 5% |
| Group | 2 | 0.00028 | 0.00014 | 0.29509 | 5.39 | 3.32 |
| AxB combination | 15 | 1,03245 | 0.06883 | 143.14 ** | 2.7 | 2.01 |
| Chicken Fertilizer | 3 | 0.64593 | 0.21531 | 447.63 ** | 4.51 | 2.92 |
| SP - 36 | 3 | 0.37364 | 0.12455 | 259,009 ** | 4.51 | 2.92 |
| Interaction | 9 | 0.01288 | 0.00143 | 2.98 * | 3.07 | 2.21 |
| Error | 32 | 0.01539 | 0.00048 |  |  |  |
| Total | 47 | 1,04813 | KK | | 3,531 | |

Note: ** = Very Real Impact

***** = Has Real Effect

**Observation of Cob Weight Without Weight (ton ha-1)**

| Combination Treatment | | Deuteronomy | | | amount | Average |
| --- | --- | --- | --- | --- | --- | --- |
| Fertilizer  Chicken Cage (A) | SP-36 (B) | 1 | 2 | 3 |  | (ton / ha) |
| P0 | S0 | 3,5388 | 3,6478 | 3,8958 | 11,0824 | 3.69 |
|  | S1 | 4,1704 | 4,7616 | 4,3812 | 13,3132 | 4.44 |
|  | S2 | 4,9715 | 4,6729 | 4,8471 | 14,4915 | 4.83 |
|  | S3 | 5,4617 | 5,3526 | 5,1732 | 15,9875 | 5.33 |
| P1 | S0 | 4,5712 | 4,4856 | 4,5028 | 13,5596 | 4.52 |
|  | S1 | 4,8094 | 4,9176 | 4,6882 | 14,4152 | 4.81 |
|  | S2 | 5,4729 | 5,2164 | 5,1387 | 15,828 | 5.28 |
|  | S3 | 5,5919 | 5,4828 | 5,3146 | 16,3893 | 5.46 |
| P2 | S0 | 4,6728 | 4,5682 | 4,7527 | 13.9937 | 4.66 |
|  | S1 | 5,2381 | 5,1056 | 5,1984 | 15,5421 | 5.18 |
|  | S2 | 5,6718 | 5,5224 | 5,7325 | 16,9267 | 5.64 |
|  | S3 | 5,7168 | 5,8197 | 5,6988 | 17,2353 | 5.75 |
| P3 | S0 | 4,9824 | 4,7933 | 4,9896 | 14,7653 | 4.92 |
|  | S1 | 5.3274 | 5,6158 | 5,5368 | 16.48 | 5.49 |
|  | S2 | 5.9371 | 5,7612 | 5,6711 | 17,3694 | 5.79 |
|  | S3 | 6,6293 | 6,1274 | 6,5889 | 19,3456 | 6.45 |
| amount |  | 82.7635 | 81,8509 | 82.1104 | 246,725 | 5.14 |

**Table of Check Variety of Cob Weights Without Weight**

| Source of Diversity | Level | amount | Square | F | F Table | |
| --- | --- | --- | --- | --- | --- | --- |
|  | Free | Square | Middle | Count | 1% | 5% |
| Group | 2 | 0.02764 | 0.01382 | 0.58458 | 5.39 | 3.32 |
| AxB combination | 15 | 19,4639 | 1,29759 | 54.8875 ** | 2.7 | 2.01 |
| Chicken Fertilizer | 3 | 7,67008 | 2,55669 | 108,147 ** | 4.51 | 2.92 |
| SP - 36 | 3 | 11,154 | 3,71799 | 157,269 ** | 4.51 | 2.92 |
| Interaction | 9 | 0.63986 | 0.0711 | 3,0073 * | 3.07 | 2.21 |
| Error | 32 | 0.75651 | 0.02364 |  |  |  |
| Total | 47 | 20,248 | KK | | 1,043 | |

Note: ** = Very Real Impact

***** = Has Real Effect

**Correlation Regression Table pH, P-Total, P-Available, P Uptake and Cob Weight Without Weight**

|  |  | pH | P-Total | P-Available | Uptake P | The weight of the cob without the weight |
| --- | --- | --- | --- | --- | --- | --- |
| pH | Pearson Correlation | 1 | 0.922 (**) | 0.930 (**) | 0.960 (**) | 0.923 (**) |
|  | Sig. (2-tailed) |  | 0,000 | 0,000 | 0,000 | 0,000 |
|  | N | 16 | 16 | 16 | 16 | 16 |
| P - Total | Pearson Correlation | 0.922 (**) | 1 | 0.972 (**) | 0.926 (**) | 0.897 (**) |
|  | Sig. (2-tailed) | 0,000 |  | 0,000 | 0,000 | 0,000 |
|  | N | 16 | 16 | 16 | 16 | 16 |
| P-Available | Pearson Correlation | 0.930 (**) | 0.972 (**) | 1 | 0.942 (**) | 0.926 (**) |
|  | Sig. (2-tailed) | 0,000 | 0,000 |  | 0,000 | 0,000 |
|  | N | 16 | 16 | 16 | 16 | 16 |
| Uptake P | Pearson Correlation | 0.960 (**) | 0.926 (**) | 0.942 (**) | 1 | 0.916 (**) |
|  | Sig. (2-tailed) | 0,000 | 0,000 | 0,000 |  | 0,000 |
|  | N | 16 | 16 | 16 | 16 | 16 |
| Cob Weight Without Weight | Pearson Correlation | 0.923 (**) | 0.897 (**) | 0.926 (**) | 0.916 (**) | 1 |
|  | Sig. (2-tailed) | 0,000 | 0,000 | 0,000 | 0,000 |  |
|  | N | 16 | 16 | 16 | 16 | 16 |

Note: ** Correlation is significant at the 0.01 level (2-tailed).

**"Step Wise" Regression Analysis**

**Variables Entered / Removed (a)**

| **Model** | **Variables Entered** | **Removed Variables** | **Method** |
| --- | --- | --- | --- |
| 1 | P - Available | . | Stepwise (Criteria: Probability-of-F-to-enter <=, 050, Probability-of-F-to-remove> =, 100). |

a Dependent Variable: The weight of the tongko without the klobot

**Model Summary**

| **Model** | **R** | **R Square** | **Adjusted R Square** | **Std. Error of the Estimate** |
| --- | --- | --- | --- | --- |
| 1 | 0.926 (a) | 0.858 | 0.848 | 0.25708 |

a Predictors: (Constant) P - Available

**ANOVA (b)**

| **Model** |  | **Sum of Squares** | **df** | **Mean Square** | **F** | **Sig.** |
| --- | --- | --- | --- | --- | --- | --- |
|  | Regression | 5,578 | 1 | 5,578 | 84,405 | 0,000 (a) |
| 1 | Residual | 0.925 | 14 | 0.066 |  |  |
|  | Total | 6,504 | 15 |  |  |  |

a Predictors: (Constant), P-Available

b Dependent Variable: The weight of the ear without the weight

**Coefficients (a)**

| **Model** |  | **Unstandardized Coefficients** | | **Standardized Coefficients** | **t** | **Sig.** |
| --- | --- | --- | --- | --- | --- | --- |
|  | | **B** | **Std. Error** | **Beta** | **B** | **Std. Error** |
| 1 | (Constant) | 2,098 | 0.337 |  | 6,219 | 0,000 |
|  | P-Available | 0.087 | 0.009 | 0.926 | 9,187 | 0,000 |

a Dependent Variable: The weight of the ear without the cob

**Excluded Variables (b)**

| **Model** | |  | **Beta In** | **t** | **Sig.** | **Partial Correlation** | **Collinearity Statistics** |
| --- | --- | --- | --- | --- | --- | --- | --- |
|  | | | **Tolerance** | **Tolerance** | **Tolerance** | **Tolerance** | **Tolerance** |
|  | pH | | 0.458 (a) | 1,805 | 0.094 | 0.448 | 0.136 |
| 1 | P-Total | | -0.055 (a) | -0,124 | 0.903 | -0.034 | 0.055 |
|  | Uptake P | | 0.385 (a) | 1,315 | 0.211 | 0.343 | 0.113 |

a Predictors in the Model: (Constant), P-Available

b Dependent Variable: The weight of the ear without the weight
