# Supplementary material for: The impact of liquefaction disaster on farming systems at agriculture land based on technical and psychosocial perspectives
Source: PLoS One. 2021 Jan 25;16(1):e0245591. doi: 10.1371/journal.pone.0245591 (PMC7834136; doi:10.1371/journal.pone.0245591)
Supplement: Attachment — Submitted filename: Reviewer Attachment.pdf [file pone.0245591.s003.pdf]

PLOS ONE

The Impact of Liquefaction Disaster on Farming Systems at Agriculture Land Based on Technical and Psychosocial Perspectives

--Manuscript Draft--

**Manuscript Number:**	PONE-D-20-00593
**Article Type:**	Research Article
**Full Title:**	The Impact of Liquefaction Disaster on Farming Systems at Agriculture Land Based on Technical and Psychosocial Perspectives
**Short Title:**	The Impact of Liquefaction Disaster on Farming Systems

Corresponding Author: Muhammad Basir-Cyio, Ph.D
Universitas Tadulako
Palu, Indonesia INDONESIA

Keywords: liquefaction; farming systems; land based; psychosocial perspectives

Abstract: This research aims to determine the attitudes of the farmers whose lands are affected by liquefaction in Jono Oge, Central Sulawesi Province. The methods used here were integrated survey and experimental design. The survey approach was intended to figure out the attitudes of the farmers viewpoints : (1) to return to their activities on the agricultural lands affected by liquefaction; (2) to consume their own agricultural products; and, (3) of their willingness to be relocated. The experimental design approach was used to figure out the effectiveness of organic material input combined with the SP-36 fertilizer. The obtained results were analyzed using the Likert Scale, diversity test, correlational test, and regression test. The results showed that the farmers persevered farming on the lands affected by liquefaction (Index = 88.82%), yet refused to consume their own agricultural products with the reason that corpses remained buried beneath their lands (Index = 27.82%); and, they also refused to be relocated (Index = 28.80 %). The continued production suitability of the affected land was also investigated. Terrain profile identification results in Jono Oge showed the disaster impact was dominantly landslide as itstill showed a clear characteristic horizon between the top soil and the sub soil. This contrasts to terrain at Petobo, Central Sulawesi Province, where the high mix of the top soil with the sub soil of agricultural land affected by liquefaction , prevented demarcation of the horizon. The land treatment of organic material and SP-36 fertilizer showed that the combined dose (M) of 40-kg ha^{-1} with P 300-kg ha^{-1} had the highest effect by changing the field pH from 5.7 to 6.41, increased the availability of P and increasedthe corncob indicator plant weight . Based on these indications, the lands affected by the liquefaction in Jono Oge can still be used as agricultural lands through restoration, from both social and technical aspects.

Order of Authors:
Muhammad Basir-Cyio, Ph.D
Mahfudz Mahfudz
Isrun Isrun
Zefitni Zefitni

Additional Information:

Question	Response
**Financial Disclosure**<br><br>Enter a financial disclosure statement that describes the sources of funding for the work included in this submission. Review the submission guidelines for detailed requirements. View published research articles from *PLOS ONE* for specific	YES<br>This research was conducted because of the funding support prepared by the Board of Professor of Tadulako University, and collaboration with Professor Timothy Roberts from the Hunter Innovation and Science Hub of the University of Newcastle, Australia. We also wish to acknowledge Melanie Ball for English editing of the manuscript.

Powered by Editorial Manager® and ProduXion Manager® from Aries Systems Corporation

examples.<br><br>This statement is required for submission and **will appear in the published article** if the submission is accepted. Please make sure it is accurate.<br><br>**Unfunded studies**<br>Enter: *The author(s) received no specific funding for this work.*<br><br>**Funded studies**<br>Enter a statement with the following details:<br>• Initials of the authors who received each award<br>• Grant numbers awarded to each author<br>• The full name of each funder<br>• URL of each funder website<br>• Did the sponsors or funders play any role in the study design, data collection and analysis, decision to publish, or preparation of the manuscript?<br>• **NO** - Include this sentence at the end of your statement: *The funders had no role in study design, data collection and analysis, decision to publish, or preparation of the manuscript.*<br>• **YES** - Specify the role(s) played.<br><br>* typeset	
**Competing Interests**<br><br>Use the instructions below to enter a competing interest statement for this submission. On behalf of all authors, disclose any competing interests that could be perceived to bias this work—acknowledging all financial support and any other relevant financial or non-financial competing interests.<br><br>This statement **will appear in the published article** if the submission is accepted. Please make sure it is accurate. View published research articles from *PLOS ONE* for specific examples.	The authors have declared that no competing interests exist.

Powered by Editorial Manager® and ProduXion Manager® from Aries Systems Corporation

**NO authors have competing interests**<br><br>Enter: *The authors have declared that no competing interests exist.*<br><br>**Authors with competing interests**<br><br>Enter competing interest details beginning with this statement:<br><br>*I have read the journal's policy and the authors of this manuscript have the following competing interests: [insert competing interests here]*<br><br>* typeset	
**Ethics Statement**<br><br>Enter an ethics statement for this submission. This statement is required if the study involved:<br><br>• Human participants<br>• Human specimens or tissue<br>• Vertebrate animals or cephalopods<br>• Vertebrate embryos or tissues<br>• Field research<br><br>Write "N/A" if the submission does not require an ethics statement.<br><br>General guidance is provided below. Consult the submission guidelines for detailed instructions. **Make sure that all information entered here is included in the Methods section of the manuscript.**	This research assigned human participants to evaluate the effect of earthquake and liquefaction to their income, health. work behavior. They have been well informed that the information they provided in the questionnaire will not affected their activities in the farmer. The study conducted based on UNTAD Reasearch Centre Contract Number: 911.f/UN28.2/PL/2019, and the Ethical Clearance Number 0014/Sinenik/UN.28/2019 from the Professor Board of Tadulako University. Consent from the farmers as participants have been obtained, and the written description and personal details of the farmer has been removed and were not published anywhere in the paper.

Powered by Editorial Manager® and ProduXion Manager® from Aries Systems Corporation

**Format for specific study types**<br><br>**Human Subject Research (involving human participants and/or tissue)**<br>• Give the name of the institutional review board or ethics committee that approved the study<br>• Include the approval number and/or a statement indicating approval of this research<br>• Indicate the form of consent obtained (written/oral) or the reason that consent was not obtained (e.g. the data were analyzed anonymously)<br><br>**Animal Research (involving vertebrate animals, embryos or tissues)**<br>• Provide the name of the Institutional Animal Care and Use Committee (IACUC) or other relevant ethics board that reviewed the study protocol, and indicate whether they approved this research or granted a formal waiver of ethical approval<br>• Include an approval number if one was obtained<br>• If the study involved *non-human primates*, add *additional details* about animal welfare and steps taken to ameliorate suffering<br>• If anesthesia, euthanasia, or any kind of animal sacrifice is part of the study, include briefly which substances and/or methods were applied<br><br>**Field Research**<br><br>Include the following details if this study involves the collection of plant, animal, or other materials from a natural setting:<br>• Field permit number<br>• Name of the institution or relevant body that granted permission	
**Data Availability**<br><br>Authors are required to make all data underlying the findings described fully available, without restriction, and from the time of publication. PLOS allows rare exceptions to address legal and ethical concerns. See the PLOS Data Policy and FAQ for detailed information.	Yes - all data are fully available without restriction

A Data Availability Statement describing where the data can be found is required at submission. Your answers to this question constitute the Data Availability Statement and **will be published in the article**, if accepted.<br><br>**Important:** Stating 'data available on request from the author' is not sufficient. If your data are only available upon request, select 'No' for the first question and explain your exceptional situation in the text box.<br><br>Do the authors confirm that all data underlying the findings described in their manuscript are fully available without restriction?	
**Describe where the data may be found in full sentences. If you are copying our sample text, replace any instances of XXX with the appropriate details.**<br><br>• If the data are **held or will be held in a public repository**, include URLs, accession numbers or DOIs. If this information will only be available after acceptance, indicate this by ticking the box below. For example: *All XXX files are available from the XXX database (accession number(s) XXX, XXX.*).<br>• If the data are all contained **within the manuscript and/or Supporting Information files**, enter the following: *All relevant data are within the manuscript and its Supporting Information files.*<br>• If neither of these applies but you are able to provide **details of access elsewhere**, with or without limitations, please do so. For example:<br><br>*Data cannot be shared publicly because of [XXX]. Data are available from the XXX Institutional Data Access / Ethics Committee (contact via XXX) for researchers who meet the criteria for access to confidential data.*<br><br>*The data underlying the results presented in the study are available from (include the name of the third party*	All relevant data are within the manuscript and its Supporting Information files.

and contact information or URL).<br>• This text is appropriate if the data are owned by a third party and authors do not have permission to share the data.<br><br>* typeset	
Additional data availability information:	Tick here if the URLs/accession numbers/DOIs will be available only after acceptance of the manuscript for publication so that we can ensure their inclusion before publication.

Manuscript
Click here to access/download;Manuscript;Manuscript Article Liquefaction in Newcastle MB TR.docx

The Impact of Liquefaction Disaster on Farming Systems at Agriculture Land Based on Technical and Psychosocial Perspectives

Muhammad BASIR-CYIO
Department of Agrotechnology, Faculty of Agriculture,
Tadulako University, Indonesia
basircyio@yahoo.com
MAHFUDZ
Department of Agrotechnology, Faculty of Agriculture,
Tadulako University, Indonesia
mahfudzuntad@gmail.com
ISRUN
Department of Agrotechnology, Faculty of Agriculture,
Tadulako University, Indonesia
isrunbaso@gmail.com
ZEFITNI
Department of Geology, Faculty of Engineering,
Tadulako University, Indonesia
zefitni_04@yahoo.com

Corresponding author: Muhammad Basir-Cyio (basircyio@yahoo.com & basircyio@untad.ac.id)

Abstract

This research aims to determine the attitudes of the farmers whose lands are affected by liquefaction in Jono Oge, Central Sulawesi Province. The methods used here were integrated survey and experimental design. The survey approach was intended to figure out the attitudes of the farmers viewpoints : (1) to return to their activities on the agricultural lands affected by liquefaction; (2) to consume their own agricultural products; and, (3) of their willingness to be relocated. The experimental design approach was used to figure out the effectiveness of organic material input combined with the SP-36 fertilizer. The obtained results were analyzed using the Likert Scale, diversity test, correlational test, and regression test. The results showed that the farmers persevered farming on the lands affected by liquefaction (Index = 88.82%), yet refused to consume their own agricultural products with the reason that corpses remained buried beneath their lands (Index = 27.82%); and, they also refused to be relocated (Index = 28.80 %). The continued production suitability of the affected land was also investigated. Terrain profile identification results in Jono Oge showed the disaster impact was dominantly landslide as itstill showed a clear characteristic horizon between the top soil and the sub soil. This contrasts to terrain at Petobo, Central Sulawesi Province, where the high mix of the top soil with the sub soil of agricultural land affected by liquefaction , prevented demarcation of the horizon. The land treatment of organic material and SP-36 fertilizer showed that the combined dose (M) of 40-kg ha^{-1} with P 300-kg ha^{-1} had the highest effect by changing the field pH from 5.7 to 6.41, increased the availability of P and increasedthe corncob indicator plant weight . Based on these indications, the lands affected by the liquefaction in Jono Oge can still be used as agricultural lands through restoration, from both social and technical aspects.

***Keywords*:** liquefaction; farming systems; land based; psychosocial perspectives.

Introduction

The earthquake, liquefaction, and tsunami, which simultaneously shook the city of Palu, Donggala Regency, and Sigi Regency, Central Sulawesi Province, Indonesia, on September 28, 2018, destroyed infrastructure, and killed no less than 10 thousand people, even more. The - number of lost lives reported by the government is based on recovered bodies. The number of victims still buried due to liquefaction and those carried by tsunami waves is difficult to detect because the events are unexpected, and in complete dark conditions. There are an unknown number of victims in addition to former residents of the city of Palu, Sigi and Donggala, as many visitors were at the city of Palu to witness the *Nomoni* traditional event. This local term describes the practise of giving of offerings to the gods around Palu Bay, as a sign of gratitude for the peace of enduring cultural preservation.

Three types of natural disasters that simultaneously occurred, in addition to stopping all economic activity, have also changed the legal aspects of agricultural land ownership affected by the faction. This is due to a shift and displacement of the position of agricultural land between two to three kilometers from the initial position before there was disturbance, including cultivated plants on the land. The shifted geographical locations require new studies and regulations from the government so land ownership and condition can be technically accountable. Earthquakes that occur everywhere will have physical, social, and economic impacts (Brante et al. 2019; Subedi and Bahadur Poudyal Chhetri 2019). In fact, traumatic conditions will overshadow people's lives (Crane et al. 2016), including apathy which threatens to decrease the creativity of the affected people (de Bruijn and Janssen 2017).

During the nearly seven months since the natural disaster occurred, there has been no farmer activity on the lands being cleared of disaster debris. There are several factors why farmers are still reluctant to plant crops on land previously used as an agricultural area. There are doubts over the condition of the land under faction which is considered no longer suitable for agriculture. The legal and technical aspects need resolution. There is also the hindering view the damaged land still holds many corpses of victims of liquefaction. Trauma is a physical disorder that takes a long time to heal (Spitzer et al. 2009), and on the other hand, farmers are pressured to meet immediate daily needs such as food. Traumatic conditions have a direct impact on the attitude of victims of whatever type of disaster they experienced (Ponnamperuma and Nicolson 2018), including decision makers (Cieslak et al. 2014). This condition will have a long-term impact on local socio-economic aspects, especially poverty and declining economic transactions (Grzenda 2019; Vartanian et al. 2006). The agricultural sector must continue to produce both rice and corn agricultural products which society needs for family consumption. In a traumatic transition period, victims need to be encouraged to have a strong vision for their future or a compelling the vision for the future (Papulova 2014).

The principles of sustainable development in the agricultural sector must continue to be considered without reducing concern for safety and environmental sustainability by promoting the value of humanity (Muschett 2017). The essence of sustainability is the ability to guarantee Humanity by continuing to get what is needed today without compromising the rights of future generations to continue to get their needs met (Seymour 2016). Ongoing local population security relies on productive agricultural land resources that were affected by liquefaction in Central Sulawesi. The faction of agricultural land which was affected by the liquefaction has experienced a very drastic change, both from environmental and socio-economic aspects. This condition has an impact on farmers' psychocultural needs of spirit, requiring remedies from both scientists and the government so that survivor farmers can continue their key socio-economic roles while maintaining environmental sustainability.

The aim of the scientists' study is to provide information from the results of research while at the same time demonstrating impacts of farming practices. Concurrently local governments provide guarantees that local land ownership rights remain a concern of the government that is being resolved through persuasive socialization. It is expected that agricultural production results from convoluted land can be accomplished, both for farmer families' consumption and for sale to traditional markets in order to obtain education costs for their children. The three main components of sustainability include the community (farmers), economy, and the environment, combined for support by government policies with scientific considerations of research results, are the pillars of strengthening sustainable development (Magee, Scerri, and James 2012; Manurung, Basir-Cyio, and Basri 2019).

Based on these reasons, a series of studies have been carried out and the results can be disseminated to the farmers and their families to obtain the correct information as well as to commence the revival of their occupational activities.

Materials and Methods

This research was carried out on the land affected by liquefaction caused by the earthquake in Jono Oge, Sigi Regency, by taking a comparison to the liquefaction-affected land area in Petobo, Palu, Central Sulawesi. The research began in April to June 2019, when the farmers who were the victims of liquefaction had occupied the temporary housings, built by the government and with assistance by the international NGOs. The area of Petobo agricultural land affected by the earthquake was around 457.10 ha, to the extent the farmers were totally failed in harvesting, while the liquefaction-affected agricultural land in Jono Oge was around 124.20 ha. The map of the research location is presented in Figure 1.

This research assigned human participants to evaluate the effect of earthquake and liquefaction to their income, health. work behavior. They have been well informed that the information they provided in the questionnaire will not affected their activities in the farmer. The study conducted based on UNTAD Reasearch Centre Contract Number: 911.f/UN28.2/PL/2019, and the Ethical Clearance Number 0014/Sinenik/UN.28/2019 from the Professor Board of Tadulako University. Consent from the farmers as participants have been obtained, and the written description and personal details of the farmer has been removed and were not published anywhere in the paper.

Figure 1. Locations of research in Jono Oge and Petobo, Central Sulawesi

The Jono Oge agricultural area is the focus of the research, while the Petobo agricultural land is only a comparison, because the two liquefaction patterns between Jono Oge and Petobo are not the same from the perspective of the workable land use for agricultural activities.

Survey Approach

The field survey was carried out in two strategic segments, namely regarding human beings, in this case, the farmers affected by natural disasters, and the condition of the agricultural land environment, where the farmers make a living and financial resources for their families, and the education costs of their children. The determination of the number of the

liquefaction-affected respondent farmers used the Slovin Formula, mathematically presented as follows:

$$n = N/\{(1 + N(e)^2\}$$

Information: n = number of Samples
N = Total Population
e = Error Tolerance Limit

Based on this equation, 34 respondents or around 10% of 340 population of farmers were affected by the earthquake and liquefaction. The respondents were sampled from those who worked in Jono Oge, while those who tried to farm in Petobo were not interviewed and no soil sampling was conducted. The two locations show different patterns of land disturbance. Liquefaction in Jono Oge is dominantly "landslide" so that the layer, or horizon, in the soil profile tends not to change to the depth of the solum 0.50 to 2.50 meters. Different results are found in the liquefaction in Petobo, where the pattern and composition of the profile layer undergoes very drastic change, where the top soil and sub-soil of the original soil cannot be identified by its texture, color, or root system.

The in-depth interviews were designed to demonstrate three attitudes of respondent farmers, namely (i) the wish to be back on planting in the liquefaction-affected land; (ii) the willingness to consume agricultural products on the liquefaction-affected land; and (iii) the responses to the government's plan to relocate elsewhere. The farmers' responses to these three attitudes and decisions are identified on Likert Scale (Joshi *et al.*, 2015) with a range of perceptions, "Strongly disagree-1–2–3–4–5 – Strongly agree". By paying attention to the statement items, the respondent farmers affected by liquefaction disaster had to choose numbers 1 to 5 based on the following criteria:

1. strongly agree = 5
2. agree = 4
3. somewhat agree = 3
4. disagree = 2
5. strongly disagree = 1

The determination of the attitudes of the respondent farmers is presented in the Index (%) which is the value of the total score divided by the maximum score times 100 percent [I = (TS/MS) x 100%]. Assessment Intervals are presented in an index range of 0.00% - 19.99% (Strongly Disagree); 20.00% - 29.99% (Disagree); 40.00% - 59.99% (Somewhat Agree); 60.00% - 79.99% (Agree) and 80.00% - 100.00% (Strongly Agree).

The perceptional data from the respondent farmers affected by natural disasters are strategic facts to be integrated with the data on the assessment of agricultural land resources, a place where farmers obtain a source of life in managing their families, both to meet their daily needs, and related to the sustainability of their children's education. The implementation of the assessment of the agricultural lands affected by the liquefaction disaster, was carried out with three approaches, namely; (i) the soil profile analysis, in order to obtain any information on the topsoil to subsoil conditions, the layers that determine the suitability of the soil to provide the carrying capacity for agricultural activities, according to the type of plant being developed; (ii) the analysis of soil samples in the laboratory, to find out in detail the physical-chemical properties of the soil; and (iii) the direct planting on the liquefaction-affected lands, and by taking soil samples to be assessed in the green house as a comparison of the

visualization of the biological condition of the vegetative plant growth with the vegetative growth planted directly on the field.

Experimental Design

The field treatment experiments applied chicken manure fertilizer and TSP-36 fertilizer with the aim of improving the physical condition of the liquefaction-affected soil, using the experimental design method. The experimental method (Seltman, 2018) determines the effectiveness of chicken manure fertilizer (M) and phosphorus fertilizer (SP-36) which contain P_2O_5 (36%). An initial soil sample assessment indicated that the soil was less fertile. The experimental land was set in the form of 1 m x 3 m (3 m^2) plots. The chicken manure (M) consisted of four dosage levels, namely (i) M_0 (0 t ha^{-1} is equivalent to 0 kg $plot^{-1}$); M_1 (10 t ha^{-1} is equivalent to 5 kg $plot^{-1}$), M_2 (25 t ha^{-1} is equivalent to 12.5 kg $plot^{-1}$) and M_3 (40 t ha^{-1} is equivalent to 20 kg $plot^{-1}$). The test applications of SP-36 synthetic fertilizer consisted of four dosage levels of S_0 (0 kg ha^{-1} equivalent to 0 g map^{-1}), S_1 (100 kg ha^{-1} equivalent to 50 g $plot^{-1}$), S_2 (200 kg ha^{-1} is equivalent to 100 g $plot^{-1}$) and S_3 (300 kg ha^{-1} SP-36 is equivalent to 150 g $plot^{-1}$). There were 16 combinations of treatments, namely: M_0S_0; M_0S_1; M_0S_2; M_0S_3; M_1S_0; M_1S_1; M_1S_2; M_1S_3; M_2S_0; M_2S_1; M_2S_2; M_2S_3; M_3S_0; M_3S_1; M_3S_2; and M_3S_3. All treatment combinations were repeated three times so that the total experimental units were 48 plots. The observed variables included the soil physical-chemical properties, bulk density, P total, P available, dry weight of plant, phosphorus uptake, and weight of skinless cob.

Data Analysis

(i) Likert Scale data analysis

The data analyzed from the interviews and questionnaires were tabulated after the respondent farmers selected numbers 1 to 5 on the Likert Scale. The Likert Score Total calculation results for the first statement (i), "The farmers' wish to return to their activities on the liquefaction-affected lands," obtained the total score (TS) = the total number of total subtotals of each answer choice, from a maximum score of 340 and a minimum score of 34. The Maximum Score = 34 x 5 = 170 (number of respondents x highest Likert Score) and The Minimum Score = 34 x 1 = 34 (number of respondents x lowest Likert Score). Index Calculation (%) = (Total Score/Maximum Score) x 100%. For the statement (i), "The wish to return for planting on the liquefaction-affected land," can be seen in the following results:

The answer of *strongly agree* = 19 respondents x 5 = 95
The answer of *agree* = 10 respondents x 4 = 40
The answer of *somewhat agree* = 4 respondents x 3 = 12
The answer of *disagree* = 2 respondents x 2 = 4
The answer of *strongly disagree* = 0 respondent x 1 = 0

Total Score = 151, or Index (151/170) x 100% = 88.8, was between the 00.00% - 100% assessment interval which means that the farmers "Very Agree" to return to their activities on their liquefaction-affected lands. The same calculation pattern was also carried out for the other two statements. For the statement (ii), the wish to consume agricultural products of the liquefaction-affected lands obtained TS = 46, or Index (46/170) x 100% = 27.06%, was between the assessment intervals of 20.00% - 39.99% which means that the farmers "Disagree" to consume the agricultural products obtained from the liquefaction-affected agricultural lands. Meanwhile, for the statement (iii) about the willingness to be relocated, the

TS = 49, or Index (49/170) x 100% = 28.82%, was between the assessment intervals of 20.00% - 39.99% which means that the farmers "Do not Agree” to be relocated to other places, or they still wanted to survive on the liquefaction-affected lands to conduct farming.

(ii) Field Assessment, Soil Sample Analysis, and Field Experiments

The field assessment was focused on identifying the soil profiles through visual observation of the horizon or the ground layer. In assessing the soil profile, the changes in some physical properties of the soil were carefully examined, especially the changes in the layers due to landslide or liquefaction. There were three soil profiles identified, so that the differences of the layer change pattern in Jono Oge and those around Petobo can be explained. The land in Jono Oge generally experienced landslide or body soil (topsoil - subsoil) position movement to a new coordinate point. In the shifting process, the mix between the topsoil and subsoil layers was minimal, while the agricultural land in Petobo was dominated by the horizon mix process, making observations indicatively difficult to distinguish between the initial topsoil and subsoil because the boundaries between the two layers were visually unidentifiable.

Soil physical-chemical properties were determined through laboratory analysises of composite soil samples on the liquefaction-unaffected land and the liquefaction-affected land, using the Lab Procedures and Methods developed by the University of Wisconsin-Extension-Madison (Schulte, Peters and Hodgson, 2013).

The observation data of the experimental results were analyzed based on the diversity analysis. When the Fisher-test results showed a measurable treatment effect, then the Duncan's multiple range test followed.. The statistical model used was a treatment combination test, and was also presented in a regression analysis (Steel and Torrie, 1993) using SPSS software and SAS Program version 9.1. The mathematical model of multiple and linear regression was (i) [$Yij = \mu + \alpha i + \beta j + (\alpha\beta)\ ij + \varepsilon ij$], and the linear regression analysis was (ii) [$Y = a + b\mathrm{X}^2$]

Result And Discussion

A. Perception

The analyzed data from the results of interviews and questionnaires were tabulated by considering the index value (NI) to simplify determining the conclusions or perceptions of the respondent farmers whose agricultural lands were affected by liquefaction. By paying attention to the statement items, the respondents had to choose numbers 1 to 5. The total Likert Score can be seen from the calculation of each part which was assessed, as presented in Table 1.

Table 1. The Scoring Results of the Likert Value, Index, and the Attitudes of the Respondent Farmers

No.	Perceptional Scope	Score		Total Score	Indexed (%)	Assessment Interval (%)	Farmers’ Attitude
		Min	Max				
1	Wish to Return to the Activities on the Lands	34	170	151	88.80	0-19.99 (SDA)<br>20-39.99 (DA)<br>40-59.99 (SA)<br>60-79.99 (A)	Strongly Agree (SA)

	Affected by Liquefaction					80-100 (SA)	
2	Consuming the Agricultural Products from the Lands Affected by Liquidation	34	170	46	27.06	0-19.99 (SDA)<br>20-39.99 (DA)<br>40-59.99 (SA)<br>60-79.99 (A)<br>80-100 (SA)	Disagree (DA)
3	Resettlement to the locations which are not affected by natural disasters	34	170	49	28.82	0-19.99 (SDA)<br>20-39.99 (DA)<br>40-59.99 (SA)<br>60-79.99 (A)<br>80-100 (SA)	Disagree (DA)

From the tabulation of Likert Scale and Index by observing the intervals of the respondents' attitude assessment of the three attitude statements, it is revealed that the farmers who have been trying to farm on the liquefaction-affected lands strongly agreeto continue to manage their own agricultural lands, even though it has been affected by liquefaction (88.82%). The farmers still hope to be able to return to work their liquefaction-affected agricultural lands. This attitude shows that sociocultural has the power to influence one's personal decisions (Marginson and Dang 2017; Vågan 2011), including the farmers who are affected by the liquefaction natural disasters in Jono Oge. Meanwhile, the statements regarding the willingness to consume the agricultural products obtained from the liquefaction-affected agricultural lands, they said they did not agree, yet wanted to sell to the markets (Index of 27.08%). The reluctance to consume agricultural products is based on the consideration of the need for money so that it tends to be sold or because there is a wrong perception (Mowbray and Mowbray 2010; Trevelline et al. 2019). The reluctance to consume agricultural products on the liquefaction-affected lands is caused by the misperception human corpses still buried beneath the ground have the potential to be absorbed by plant roots. This attitude reflects the low-level knowledge of the farmers. A person's education can affect the mindset and also the rationality in seeing a problem (Dweck and Yeager 2019; King 2017). The third attitude is to refuse the resettlement to another safer location. This is shown by the obtained Index at 28.82% (Disagree) to leave the location adjacent to the liquefaction-affected lands. The cultural power is very decisive in social cohesion (socio-cohesion) which applies in a particular community (Auta 2013; Mesra, Erianjoni, and Eriyanti 2018).

B. Observation Data Profile and Soil Chemical-Physical Properties

The liquefaction of agricultural lands in Jono Oge and Petobo (Figure 2) has completely stopped the agricultural activities which have affected the plight of the farmers. For the liquefaction victims who have survived natural disasters, it has been difficult to obtain a livelihood because they depend only on agricultural production.

Figure 2. The liquefaction-affected agricultural areas of Jono Oge and Petobo, Central Sulawesi

The current condition of the liquefaction-affected agricultural lands is still neglected, and there has yet been no substantial agricultural activity. The farmers doubt starting to work on their agricultural lands due to a sense of trauma, furthermore, the resolve of the government is also needed. The farmers also need scientific information from researchers, both in terms of soil fertility and groundwater conditions (Zefitni, *et al.*, 2019: (Sharma et al. 2013), to ensure that the liquefaction-affected lands can still be used for planting. The profile observation results on the liquefaction-affected land in Jono Oge show that the soil has a horizon position which is still relatively the same between the liquefaction-unaffected Entisol layers. The liquefaction-affected soil in Jodo Oge (Figure 3-B) still has features which are not different from the landslide-unaffected soil (Figure 3-A), except for the only slightly gray color in the landslide lands. Some landslide processes are randomized, yet some move to a certain depth without mixing (Akgun 2012; Fan, Henriksen, and Porter 2018). The analytical results of the subsoil physical properties in the two profiles do not differ, especially in the texture composition (Table 2). The soil texture can be used as an indicator in assessing the soil physical properties (Groenendyk et al. 2015; Salley et al. 2018). The comparison results of the soil profiles have found no significant distinguishing characteristics, ranging from topsoil to the 2.5-m solum. The color changes in the landslide-affected lands indicate an infiltration of organic compounds from the soil surface (layer 0) into the topsoil, during the landslide event. The infiltration that carries the organic compounds affects the soil color changes in the subsoil, especially in the illuviation layer (Celestina et al. 2019).

Figure 3. The Profile of the liquefaction-affected soil (Landslide-B), and the Profile of the liquefaction-unaffected soil (Landslide-A)

The similarity of the soil physical properties, especially the texture and position of the topsoil and subsoil in the liquefaction-affected soil and the unaffected one, shows strong cementation between the particles in topsoil and subsoil. Cementation is a determining factor in the strength of interparticle adhesion force work (Haddad, Rocha, and Assunção 2011; Lorenzetti et al. 2019). The movements and shifts that occur in the uncemented layer underneath 2.5 meters, where the level of water saturation is maximum, do not affect the topsoil layer on the land that can be used for planting. Thus, technically it is still possible to be processed to produce agricultural products. This is also strengthened by the assessment results of the soil chemical properties in the laboratory which show no significant difference between the landslide-affected soil and the landslide-unaffected soil (Table 2).

Table 2. The Analysis of the Physical-Chemical Properties of the Initial Soil Samples

No.	Parameter	Unit	Score	Information

1	Sand	%	60.9	
2	Loam	%	10.2	Loamy clay sand
3	Clay	%	28.9	
5	Bulk density	g/cm^3	1.6	
7	C-organic	%	1.02	Low
8	N-Total	%	0.16	Low
9	C/N		8.5	Low
10	pH H_2O (1:2.5)		5.8	Somewhat Acid
11	pH KCl (1:2.5)		4.9	
12	P_2O_5 (HCl 25%)	mg/100g	24.08	Low
13	P_2O_5 (Bray I)	Ppm	10.33	Low
14	K_2O (HCl 25%)	me/100g	29.41	Low
15	Ca^{2+}	me/100g	4.65	Low
16	Mg^{2+}	me/100g	0.43	Low
17	K^+	me/100g	0.25	Low
18	Na^+	me/100g	0.17	Low
19	KTK	me/100g	23.27	Low
20	KB	%	23.63	Low
21	Al-dd	me/100g	0.35	
22	H-dd	me/100g	0.3	

Information: Analyzed at the Agrotechnology Laboratory of the Faculty of Agriculture, Tadulako University (2019)

In Table 2, it can be seen that the soil texture class is in the category of "loamy clay sand" with the composition of 60.90% sand, 10.20% dust, and 28.90% clay. The bulk density (ρ) of the soil is 1.60 g cm^{-3}. The sandy texture class has high porosity so that the water holding capacity is low (Blanco-Canqui 2017; Bodner, Leitner, and Kaul 2014). The analysis results of several chemical properties, namely the pH (H_2O) is 5.33, and the pH (KCl) is 4.66, the Al_{ex} content is 0.35 me/100 g, with the low exchangeable base ion which are Ca^{2+} (4.65 me)/100 g), Mg^{2+} (0.43 me/100 g), K^+ (0.25 me/100 g), and Na^+ (0.17 me/100 g). The N-total value is low (0.16%), as well as the C-organic (1.02%). The C/N ratio is low, the P-available is low (10.30 ppm), the P-total is moderate (24.08 mg / 100 g), the K-total is moderate (29.41 mg/100 g), and the KTK (23.27 me/100 g) is low. Based on those soil properties, the liquefaction-affected lands have low fertility, both before and after the liquefaction disaster. This condition shows that the liquefaction-affected lands from the physical aspect can still be used as agricultural areas, however it requires input to increase the nutrient availability in order to optimize the soil health and the carrying capacity for the plant growth. The administration of the organic materials input can improve the soil structure and the water holding capacity (KTK) (Bhogal et al. 2018; Ochoa-Hueso et al. 2019).

Providing input from organic material (manure) and SP-36 Fertilizer also improves the propagation chemical properties of the soil, especially pH, available P, dry weight of plant tissue, uptake of P and also the weight of corn cobs. The experimental results showed that all doses of organic fertilizer and SP-36 and their combination had a significant effect on the observed variables (Table 3). Organic matter, in addition to being a source of OH-1 groups, can also release other organic compounds (El Nady et al. 2015).

Table 3. Results of Analysis of Variance Effect (F) of Organic Fertilizers and SP-36 Fertilizers on Variable Dependents

Sources of Variants	F-calculated					
	pH-H_2O	P-total (mg/100-g soil	P-available (ppm)	Dry weight plant tissue (g $plot^{-1}$)	P-uptake (mg Kg^{-1})	Corncob Weight (g $plot^{-1}$)
Group	0.490306	2.493292	2.493292	0.667025	0.29509	0.58458
Interaction (MxP)	1327.326**	634022.2 **	634022.2 **	2931.163 **	143.14 **	54.8875 **
Manure (M)	4688.13**	2237699**	2237699**	9064.184 **	447.63 **	108.147 **
Fertilizer SP-36 (P)	1898.6**	820924.2 **	820924.2**	4948.169 **	259.009 **	157.269 **

** Treatment is significant at the 0.01 level (2-tailed).

The combination of the organic materials and the SP-36 significantly affects the physical-chemical properties of the liquefaction-affected soil. The used dosage is in accordance with the organic materials availability and the farmers availability who are dispersed by natural disasters in purchasing the SP-36 fertilizers which are easily obtained in the market. The treatments required for the organic fertilizer and the SP-36 are presented in Figure 4. The autocorrelation between the dependent variables (Table 4) shows the nutrients released from the organic fertilizer and the SP-36 into the rhizosphere adopted by the plant roots into the tissue, influence each other in the plant growth process.. The interaction between the organic materials and the synthetic fertilizer accelerates the nutrients availability required by the growth of vegetative and generative plants (Mahal et al. 2019; Saha et al. 2018).

Table 4. The correlation analysis results of pH-H_2O, P-Total, P-Available, P Absorption, and the Weight of Cobs.

Variable Indicators		pH-H_2O	P-total	P-available	P-absorption	Corncob Weight
pH-H_2O	Pearson Correlation	1	0.922(**)	0.930(**)	0.960(**)	0.923(**)
	Sig. (2-tailed)		0.000	0.000	0.000	0.000
	N	16	16	16	16	16
P- total	Pearson Correlation	0.922(**)	1	0.972(**)	0.926(**)	0.897(**)
	Sig. (2-tailed)	0.000		0.000	0.000	0.000
	N	16	16	16	16	16
P-available	Pearson Correlation	0.930(**)	0.972(**)	1	0.942(**)	0.926(**)
	Sig. (2-tailed)	0.000	0.000		0.000	0.000
	N	16	16	16	16	16
P-uptake	Pearson Correlation	0.960(**)	0.926(**)	0.942(**)	1	0.916(**)
	Sig. (2-tailed)	0.000	0.000	0.000		0.000
	N	16	16	16	16	16
Corncob Weight	Pearson Correlation	0.923(**)	0.897(**)	0.926(**)	0.916(**)	1
	Sig. (2-tailed)	0,000	0,000	0,000	0.000	
	N	16	16	16	16	16

** Correlation is significant at the 0.01 level (2-tailed).

The administration of organic fertilizer and SP-36 fertilizer can significantly increase the soil pH (H_2O). The increased soil pH (H_2O), due to chicken manure and SP-36 fertilizer, is optimum at the combination of 40-t ha^{-1} (P_3) dosage of organic fertilizer, and 300-kg ha^{-1} (P_3S_3) dosage of SP-36 fertilizer. Before being treated, the soil pH (H_2O) of 5.35 increased to 6.38, or increased by 1.04 points after treatment. The changes in pH from slightly acidic to neutral condition affect the macro nutrients availability, such as P, K, and Ca, and suppress their solubility, such as Al^{3+}, Fe^{3+}, and Mn^{2+}. The dynamics of the soil chemical properties occur due to the release of hydroxyl ions (OH^{-1}) from the decomposition of the organic materials, as a counterweight to the dynamics of H^+ ions in the soil solution (Dhaliwal et al. 2019; von der Heyden and Roychoudhury 2015). The presence of OH^- ions will stabilize the activity of H^+ ions so that the acidity of the soil is controlled under conditions which are somewhat neutral to neutral (Cherian and Arnepalli 2015). Under the neutral pH conditions, the micro nutrient ions which have the potential to fix the macro nutrient ions are also

controlled (Dimkpa and Bindraban 2016), thus the nutrients availability becomes high so that it is easily absorbed by the plant roots (Ahmad, Imran, and Hussain 2018). The adequacy of macro and micro nutrients needed by plants depends on their availability in the soil solutions around the rhizosphere (Etienne et al. 2018). The higher the availability, the more potential the root absorption process will also occur (Le Deunff and Malagoli 2014). The accumulation of nutrients in the ribosome simplifies the distribution from xylem to phloem after undergoing a process of synthesis in the plant leaf tissue cells (Zhou et al. 2015). This condition certainly still occurs in the liquefaction-affected agricultural lands so that the corn plants, as the indicator plants, grow normally due to the carrying capacity of the soil physical-chemical properties after receiving input from the organic fertilizer and SP-36 fertilizer as shown in Figure 4.

Figure 4. The impact of dosage interaction between the organic materials with the SP-36 Fertilizer on (1) pH (H_2O), (2) P-total, (3) P-available, and (4) the Weight of corncobs

The administration of 40-t ha^{-1} and SP-36 300-kg ha^{-1} (P_3S_3) dosage of the organic fertilizer had the highest effect on increasing the soil pH, P-total, P-available, and the weight of corncobs. The significant response of plants indicated that the liquefaction-affected lands required input in increasing the nutrients availability needed by plants (He, He, and Ding 2018; Welk et al. 2019). The response difference between the treatment combination of PoSo and P_3S_3 seemed significant between both the blue line and the yellow line, for the pH (1), P-total (2), P-available (3), and the weight of corncob (4). Increasing the soil pH to the neutral condition, by administering organic materials, stabilized the dynamics of the chemical properties of the soil solution, in which the micro nutrients solubility was minimized and also reduced, thus directly increased the phosphorus nutrients availability (Odutola Oshunsanya 2019; Zhang, Wu, and Liu 2019).

Con[illegible]ion

The farmers in Jono Oge continued farming in the liquefaction-affected areas, however they were reluctant to consume their own agricultural products because they thought there were still human corpse victims of liquefaction and earthquake, beneath the surface. The liquefaction-affected dual farming areas were different in the impact patterns, with the Jono Oge dominantly being landslide, so that the identifiers between the top soil and the sub soil remained easily identified; while there had been a very high mixture on the agricultural land in Petobo, thus the horizon of the soil's profile was difficult to identify. The physical-chemical condition of the liquefaction-affected soil in Jono Oge was able to be restored by administering organic materials and the SP-36 fertilizer, however, the farmers needed the support from the three parties, namely the government related to regulations, scientists to provide dissemination of the research results, and NGO activists to provide advice or assistance including to revitalize their spirit to change for a better life in the future.

Acknowledgment

This research was conducted because of the funding support prepared by the Board of Professor of Tadulako University, and collaboration with Professor Timothy Roberts from

the Hunter Innovation and Science Hub of the University of Newcastle, Australia. We also wish to acknowledge Melanie Ball for English editing of the manuscript.

References

[1] Ahmad, Rabia Shabir, Ali Imran, and Muhammad Bilal Hussain. 2018. "Nutritional Composition of Meat." In *Meat Science and Nutrition*, edited by Muhammad Sajid Arshad. InTech. https://doi.org/10.5772/intechopen.77045.

[2] Akgun, Aykut. 2012. "A Comparison of Landslide Susceptibility Maps Produced by Logistic Regression, Multi-Criteria Decision, and Likelihood Ratio Methods: A Case Study at İzmir, Turkey." *Landslides* 9 (1): 93–106. https://doi.org/10.1007/s10346-011-0283-7.

[3] Auta, Filibus Jamaka. 2013. "Systemic Population and Family Education for National Security and Development." *Journal of Educational and Social Research*, August. https://doi.org/10.5901/jesr.2013.v3n5p135.

[4] Bhogal, Anne, Fiona A. Nicholson, Alison Rollett, Matt Taylor, Audrey Litterick, Mark J. Whittingham, and John R. Williams. 2018. "Improvements in the Quality of Agricultural Soils Following Organic Material Additions Depend on Both the Quantity and Quality of the Materials Applied." *Frontiers in Sustainable Food Systems* 2 (April): 9. https://doi.org/10.3389/fsufs.2018.00009.

[5] Blanco-Canqui, Humberto. 2017. "Biochar and Soil Physical Properties." *Soil Science Society of America Journal* 81 (4): 687. https://doi.org/10.2136/sssaj2017.01.0017.

[6] Bodner, G., D. Leitner, and H.-P. Kaul. 2014. "Coarse and Fine Root Plants Affect Pore Size Distributions Differently." *Plant and Soil* 380 (1–2): 133–51. https://doi.org/10.1007/s11104-014-2079-8.

[7] Brante, Antonio, Garen Guzmán-Rendón, Erwin M. Barría, Marie-Laure Guillemin, Iván Vera-Escalona, and Cristián E. Hernández. 2019. "Post-Disturbance Genetic Changes: The Impact of the 2010 Mega-Earthquake and Tsunami on Chilean Sandy Beach Fauna." *Scientific Reports* 9 (1): 14239. https://doi.org/10.1038/s41598-019-50525-1.

[8] Bruijn, Hans de, and Marijn Janssen. 2017. "Building Cybersecurity Awareness: The Need for Evidence-Based Framing Strategies." *Government Information Quarterly* 34 (1): 1–7. https://doi.org/10.1016/j.giq.2017.02.007.

[9] Celestina, Corinne, Jennifer L. Wood, James B. Manson, Xiaojuan Wang, Peter W. G. Sale, Caixian Tang, and Ashley E. Franks. 2019. "Microbial Communities in Top- and Subsoil of Repacked Soil Columns Respond Differently to Amendments but Their Diversity Is Negatively Correlated with Plant Productivity." *Scientific Reports* 9 (1): 8890. https://doi.org/10.1038/s41598-019-45368-9.

[10] Cherian, C., and D. N. Arnepalli. 2015. "A Critical Appraisal of the Role of Clay Mineralogy in Lime Stabilization." *International Journal of Geosynthetics and Ground Engineering* 1 (1): 8. https://doi.org/10.1007/s40891-015-0009-3.

[11] Cieslak, Roman, Kotaro Shoji, Allison Douglas, Erin Melville, Aleksandra Luszczynska, and Charles C. Benight. 2014. "A Meta-Analysis of the Relationship between Job Burnout and Secondary Traumatic Stress among Workers with Indirect Exposure to Trauma." *Psychological Services* 11 (1): 75–86. https://doi.org/10.1037/a0033798.

[12] Crane, Paul K., Laura E. Gibbons, Kristen Dams-O'Connor, Emily Trittschuh, James B. Leverenz, C. Dirk Keene, Joshua Sonnen, et al. 2016. "Association of Traumatic Brain Injury With Late-Life Neurodegenerative Conditions and Neuropathologic Findings." *JAMA Neurology* 73 (9): 1062. https://doi.org/10.1001/jamaneurol.2016.1948.

[13] Dhaliwal, S.S., R.K. Naresh, Agniva Mandal, Ravinder Singh, and M.K. Dhaliwal. 2019. “Dynamics and Transformations of Micronutrients in Agricultural Soils as Influenced by Organic Matter Build-up: A Review.” *Environmental and Sustainability Indicators* 1–2 (September): 100007. https://doi.org/10.1016/j.indic.2019.100007.
[14] Dimkpa, Christian O., and Prem S. Bindraban. 2016. “Fortification of Micronutrients for Efficient Agronomic Production: A Review.” *Agronomy for Sustainable Development* 36 (1): 7. https://doi.org/10.1007/s13593-015-0346-6.
[15] Dweck, Carol S., and David S. Yeager. 2019. “Mindsets: A View From Two Eras.” *Perspectives on Psychological Science* 14 (3): 481–96. https://doi.org/10.1177/1745691618804166.
[16] El Nady, Mohamed M., Fatma S. Ramadan, Mahmoud M. Hammad, and Nira M. Lotfy. 2015. “Evaluation of Organic Matters, Hydrocarbon Potential and Thermal Maturity of Source Rocks Based on Geochemical and Statistical Methods: Case Study of Source Rocks in Ras Gharib Oilfield, Central Gulf of Suez, Egypt.” *Egyptian Journal of Petroleum* 24 (2): 203–11. https://doi.org/10.1016/j.ejpe.2015.05.012.
[17] Etienne, Philippe, Sylvain Diquelou, Marion Prudent, Christophe Salon, Anne Maillard, and Alain Ourry. 2018. “Macro and Micronutrient Storage in Plants and Their Remobilization When Facing Scarcity: The Case of Drought.” *Agriculture* 8 (1): 14. https://doi.org/10.3390/agriculture8010014.
[18] Fan, Fan, Christian Bugge Henriksen, and John Porter. 2018. “Relationship between Stoichiometry and Ecosystem Services: A Case Study of Organic Farming Systems.” *Ecological Indicators* 85 (February): 400–408. https://doi.org/10.1016/j.ecolind.2017.10.063.
[19] Groenendyk, Derek G., Ty P.A. Ferré, Kelly R. Thorp, and Amy K. Rice. 2015. “Hydrologic-Process-Based Soil Texture Classifications for Improved Visualization of Landscape Function.” Edited by Andrew C Singer. *PLOS ONE* 10 (6): e0131299. https://doi.org/10.1371/journal.pone.0131299.
[20] Grzenda, Wioletta. 2019. “Socioeconomic Aspects of Long-Term Unemployment in the Context of the Ageing Population of Europe: The Case of Poland.” *Economic Research-Ekonomska Istraživanja* 32 (1): 1561–82. https://doi.org/10.1080/1331677X.2019.1638289.
[21] Haddad, Marcela Filié, Eduardo Passos Rocha, and Wirley Gonçalves Assunção. 2011. “Cementation of Prosthetic Restorations: From Conventional Cementation to Dental Bonding Concept.” *Journal of Craniofacial Surgery* 22 (3): 952–58. https://doi.org/10.1097/SCS.0b013e31820fe205.
[22] He, Mei, Cheng-Qiang He, and Nai-Zheng Ding. 2018. “Abiotic Stresses: General Defenses of Land Plants and Chances for Engineering Multistress Tolerance.” *Frontiers in Plant Science* 9 (December): 1771. https://doi.org/10.3389/fpls.2018.01771.
[23] Heyden, Bjorn P. von der, and Alakendra N. Roychoudhury. 2015. “Application, Chemical Interaction and Fate of Iron Minerals in Polluted Sediment and Soils.” *Current Pollution Reports* 1 (4): 265–79. https://doi.org/10.1007/s40726-015-0020-2.
[24] King, Ronnel B. 2017. “A Fixed Mindset Leads to Negative Affect: The Relations Between Implicit Theories of Intelligence and Subjective Well-Being.” *Zeitschrift Für Psychologie* 225 (2): 137–45. https://doi.org/10.1027/2151-2604/a000290.
[25] Le Deunff, Erwan, and Philippe Malagoli. 2014. “Breaking Conceptual Locks in Modelling Root Absorption of Nutrients: Reopening the Thermodynamic Viewpoint of Ion Transport across the Root.” *Annals of Botany* 114 (8): 1555–70. https://doi.org/10.1093/aob/mcu203.
[26] Lorenzetti, Camila C., Janaina F. Bortolatto, Anna T. P. R. Ramos, André L. Shinohara, Jose R. C. Saad, and Milton C. Kuga. 2019. “The Effectiveness of Glass Ionomer Cement as a Fiber Post Cementation System in Endodontically Treated Teeth.” *Microscopy Research and Technique* 82 (7): 1191–97. https://doi.org/10.1002/jemt.23268.
[27] Magee, Liam, Andy Scerri, and Paul James. 2012. “Measuring Social Sustainability: A Community-Centred Approach.” *Applied Research in Quality of Life* 7 (3): 239–61. https://doi.org/10.1007/s11482-012-9166-x.
[28] Mahal, Navreet K., William R. Osterholz, Fernando E. Miguez, Hanna J. Poffenbarger, John E. Sawyer, Daniel C. Olk, Sotirios V. Archontoulis, and Michael J. Castellano. 2019. “Nitrogen

Fertilizer Suppresses Mineralization of Soil Organic Matter in Maize Agroecosystems." *Frontiers in Ecology and Evolution* 7 (March): 59. https://doi.org/10.3389/fevo.2019.00059.
[29] Manurung, Konder, Muhammad Basir-Cyio, and Hasan Basri. 2019. "The Development and Potential Evaluation of Indonesian Lore Lindu National Park Ecotourism in Relation to the Economic Growth of the Surrounding Community." *Journal of Environmental Management and Tourism,* 8. http://dx.doi.org/10.14505/jemt.v10.2(34).11.
[30] Marginson, Simon, and Thi Kim Anh Dang. 2017. "Vygotsky's Sociocultural Theory in the Context of Globalization." *Asia Pacific Journal of Education* 37 (1): 116–29. https://doi.org/10.1080/02188791.2016.1216827.
[31] Mesra, Romi, Erianjoni Erianjoni, and Fitri Eriyanti. 2018. "The Social Meaning of Money in Social Interaction of Boarding Students." In *Proceedings of the International Conferences on Educational, Social Sciences and Technology - ICESST 2018,* 43–50. Padang: Fakultas Ilmu Pendidikan. https://doi.org/10.29210/201816.
[32] Mowbray, Joan, and Helen Mowbray. 2010. "A Consideration of the Needs of the Adult Patient with Dementia Attending for Radiotherapy and Its Impact on the Practical Consent Process." *Journal of Radiotherapy in Practice* 9 (2): 107–16. https://doi.org/10.1017/S1460396909990306.
[33] Muschett, F.Douglas. 2017. *Principles of Sustainable Development.* Edited by F. Douglas Muschett. 1st ed. Routledge. https://doi.org/10.1201/9780203742051.
[34] Ochoa-Hueso, Raúl, Manuel Delgado-Baquerizo, Paul Tuan An King, Merryn Benham, Valentina Arca, and Sally A. Power. 2019. "Ecosystem Type and Resource Quality Are More Important than Global Change Drivers in Regulating Early Stages of Litter Decomposition." *Soil Biology and Biochemistry* 129 (February): 144–52. https://doi.org/10.1016/j.soilbio.2018.11.009.
[35] Odutola Oshunsanya, Suarau. 2019. "Introductory Chapter: Relevance of Soil PH to Agriculture." In *Soil PH for Nutrient Availability and Crop Performance*, edited by Suarau Oshunsanya. IntechOpen. https://doi.org/10.5772/intechopen.82551.
[36] Papulova, Z. 2014. "The Significance of Vision and Mission Development for Enterprises in Slovak Republic." *Journal of Economics, Business and Management,* 12–16. https://doi.org/10.7763/JOEBM.2014.V2.90.
[37] Ponnamperuma, Thyagi, and Nancy A. Nicolson. 2018. "The Relative Impact of Traumatic Experiences and Daily Stressors on Mental Health Outcomes in Sri Lankan Adolescents: Mental Health Impact of Trauma and Daily Stressors." *Journal of Traumatic Stress* 31 (4): 487–98. https://doi.org/10.1002/jts.22311.
[38] Saha, Biplob K., Michael T. Rose, Vanessa N. L. Wong, Timothy R. Cavagnaro, and Antonio F. Patti. 2018. "Nitrogen Dynamics in Soil Fertilized with Slow Release Brown Coal-Urea Fertilizers." *Scientific Reports* 8 (1): 14577. https://doi.org/10.1038/s41598-018-32787-3.
[39] Salley, Shawn W., Jeffrey E. Herrick, Caitlin V. Holmes, Jason W. Karl, Matthew R. Levi, Sarah E. McCord, Cornelis van der Waal, and Justin W. Van Zee. 2018. "A Comparison of Soil Texture-by-Feel Estimates: Implications for the Citizen Soil Scientist." *Soil Science Society of America Journal* 82 (6): 1526. https://doi.org/10.2136/sssaj2018.04.0137.
[40] Seymour, Valentine. 2016. "The Human–Nature Relationship and Its Impact on Health: A Critical Review." *Frontiers in Public Health* 4 (November). https://doi.org/10.3389/fpubh.2016.00260.
[41] Sharma, Rakesh, Kelly R Biedenharn, Jennifer M Fedor, and Ashok Agarwal. 2013. "Lifestyle Factors and Reproductive Health: Taking Control of Your Fertility." *Reproductive Biology and Endocrinology* 11 (1): 66. https://doi.org/10.1186/1477-7827-11-66.
[42] Spitzer, Carsten, Sven Barnow, Henry Völzke, Ulrich John, Harald J. Freyberger, and Hans Joergen Grabe. 2009. "Trauma, Posttraumatic Stress Disorder, and Physical Illness: Findings from the General Population:" *Psychosomatic Medicine* 71 (9): 1012–17. https://doi.org/10.1097/PSY.0b013e3181bc76b5.
[43] Subedi, Shiva, and Meen Bahadur Poudyal Chhetri. 2019. "Impacts of the 2015 Gorkha Earthquake: Lessons Learnt from Nepal." In *Earthquakes - Impact, Community Vulnerability and Resilience*, edited by Jaime Santos-Reyes. IntechOpen. https://doi.org/10.5772/intechopen.85322.

[44] Trevelline, Brian K., Samantha S. Fontaine, Barry K. Hartup, and Kevin D. Kohl. 2019. "Conservation Biology Needs a Microbial Renaissance: A Call for the Consideration of Host-Associated Microbiota in Wildlife Management Practices." *Proceedings of the Royal Society B: Biological Sciences* 286 (1895): 20182448. https://doi.org/10.1098/rspb.2018.2448.

[45] Vågan, André. 2011. "Towards a Sociocultural Perspective on Identity Formation in Education." *Mind, Culture, and Activity* 18 (1): 43–57. https://doi.org/10.1080/10749031003605839.

[46] Vartanian, José Guilherme, André Lopes Carvalho, Julia Toyota, Ivonete S. Giacometti Kowalski, and Luiz Paulo Kowalski. 2006. "Socioeconomic Effects of and Risk Factors for Disability in Long-Term Survivors of Head and Neck Cancer." *Archives of Otolaryngology–Head & Neck Surgery* 132 (1): 32. https://doi.org/10.1001/archotol.132.1.32.

[47] Welk, Astrid, Erik Welk, Mathias Baudis, Jörg Böckelmann, and Helge Bruelheide. 2019. "Plant Species' Range Type Determines Local Responses to Biotic Interactions and Land Use." *Ecology*, October. https://doi.org/10.1002/ecy.2890.

[48] Zhang, Yun-Yi, Wei Wu, and Hongbin Liu. 2019. "Factors Affecting Variations of Soil PH in Different Horizons in Hilly Regions." Edited by João Canário. *PLOS ONE* 14 (6): e0218563. https://doi.org/10.1371/journal.pone.0218563.

[49] Zhou, Xiang, Wen-Juan Liao, Jun-Ming Liao, Peng Liao, and Hua Lu. 2015. "Ribosomal Proteins: Functions beyond the Ribosome." *Journal of Molecular Cell Biology* 7 (2): 92–104. https://doi.org/10.1093/jmcb/mjv014.

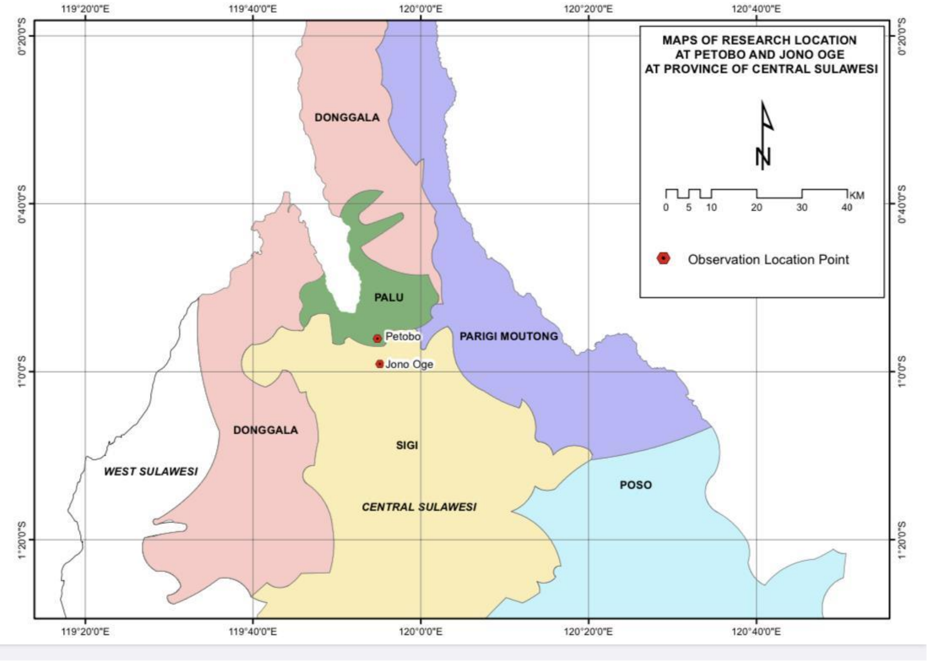

Figure 1. Locations of research in Jono Oge and Petobo, Central Sulawesi

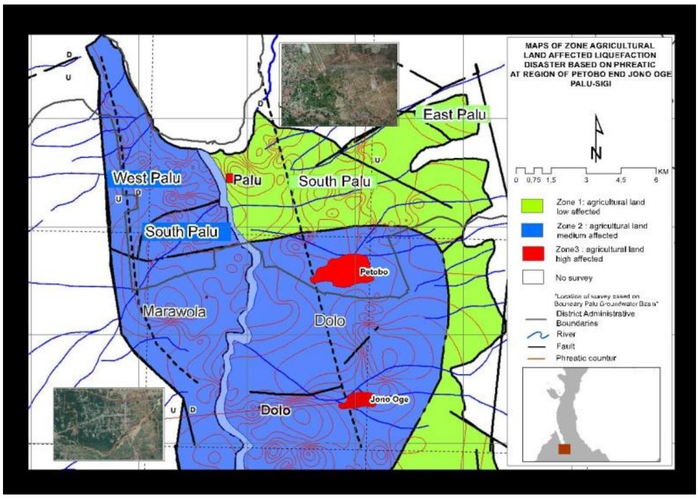

Figure 2. The liquefaction-affected agricultural areas of Jono Oge and Petobo, Central Sulawesi

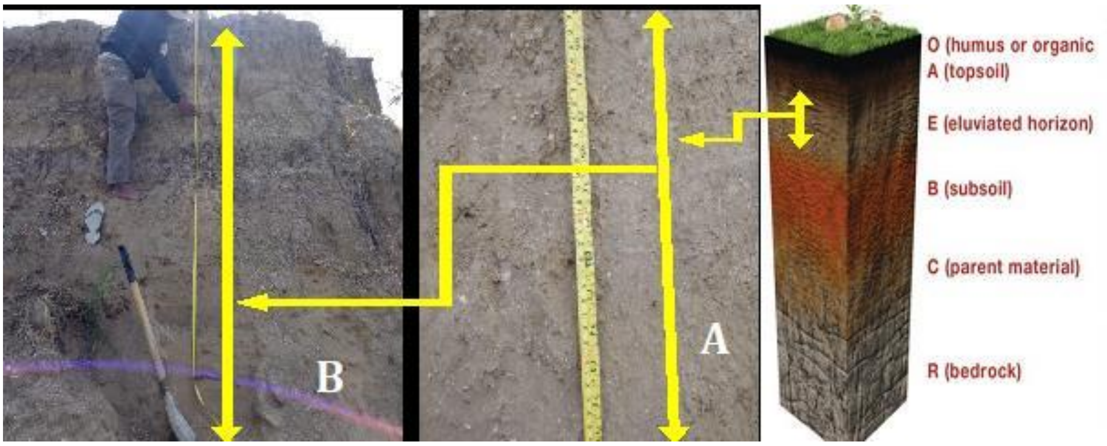

Figure 3. The Profile of the liquefaction-affected soil (Landslide-B), and the Profile of the liquefaction-unaffected soil (Landslide-A)

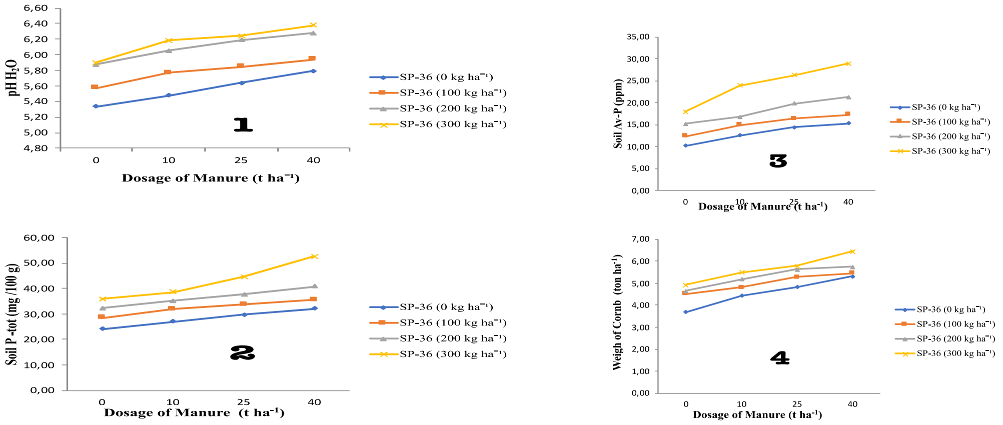

Figure 4. The impact of dosage interaction between the organic materials with the SP-36 Fertilizer on (1) pH (H_2O), (2) P-total, (3) P-available, and (4) the Weight of corncobs
