# Supplementary material for: The impact of liquefaction disaster on farming systems at agriculture land based on technical and psychosocial perspectives
Source: PLoS One. 2021 Jan 25;16(1):e0245591. doi: 10.1371/journal.pone.0245591 (PMC7834136; doi:10.1371/journal.pone.0245591)
Supplement: Attachment — Submitted filename: Reviewers Coment (1).docx [file pone.0245591.s004.docx]

Reviewers' comments:

Reviewer's Responses to Questions

**Comments to the Author**

1. Is the manuscript technically sound, and do the data support the conclusions?

The manuscript must describe a technically sound piece of scientific research with data that supports the conclusions. Experiments must have been conducted rigorously, with appropriate controls, replication, and sample sizes. The conclusions must be drawn appropriately based on the data presented.

Reviewer #2: Partly

Reviewer #3: Partly

**We have reviewed and corrected the suggestions and corrections from the Review Team. Thank you for all the help and advice.**

2. Has the statistical analysis been performed appropriately and rigorously?

Reviewer #2: No

Reviewer #3: Yes

**We have read and reviewed the Statistical Analysis in the article. From the results of the re-examination, it was found that the Statistical Analysis that we wrote was appropriate. Re-examination has been done to the maximum.**

3. Have the authors made all data underlying the findings in their manuscript fully available?

The [PLOS Data policy](http://www.plosone.org/static/policies.action#sharing) requires authors to make all data underlying the findings described in their manuscript fully available without restriction, with rare exception (please refer to the Data Availability Statement in the manuscript PDF file). The data should be provided as part of the manuscript or its supporting information, or deposited to a public repository. For example, in addition to summary statistics, the data points behind means, medians and variance measures should be available. If there are restrictions on publicly sharing data—e.g. participant privacy or use of data from a third party—those must be specified.

Reviewer #2: Yes

Reviewer #3: No

**Thank you for the advice from the Reviewer Team. In our opinion, the data underlying the findings are contained and written in the manuscript as a whole, without exception.**

4. Is the manuscript presented in an intelligible fashion and written in standard English?

PLOS ONE does not copyedit accepted manuscripts, so the language in submitted articles must be clear, correct, and unambiguous. Any typographical or grammatical errors should be corrected at revision, so please note any specific errors here.

Reviewer #2: Yes

Reviewer #3: Yes

**The language used in the manuscript is standard English which is easy to understand and unambiguous. Thank you for the review.**

5. Review Comments to the Author

Please use the space provided to explain your answers to the questions above. You may also include additional comments for the author, including concerns about dual publication, research ethics, or publication ethics. (Please upload your review as an attachment if it exceeds 20,000 characters)

Reviewer #2: The objective of the study is very broad not clearly elaborated. It doesnot specify what the study is going to investigate.
The methodology is weak. The perception survey is carried out only in one site. Two sites would be better for comparison as well as for better concrete results. The experimental methods does not indicate where and why the experiments were conducted.
Some references is missing. For instance, Joshi et al 2015 is missing.
There are many typos in the paper
Implications of research findings and limitation of the studies are not clearly outlined.

Reviewer #3: This paper is quite interesting. It takes a multi-disciplinary approach towards understanding how farmer attitudes towards continued cultivation of liquefaction-affected rural fields are shaped by likely presence of the dead, embedded in areas of landsliding and liquefaction, more than the actual soil physical-chemical properties of the soil. IN a general sense, the farmers are willing to continue to farm in these areas, and unwilling to move elsewhere, but unwilling to consume their own products grown in these fields. The authors attribute the latter to suggest that farmers are worried about 'root uptake' of the deceased into plants, and go on to make the unfortunate mistake of stating that this reflects the "low-level knowledge" of the farmers. The latter statement is arrogant and should be removed; there are many types of knowledge, including experience, and it is not impossible that degrading corpses also contribute locally to changes in soil chemistry. I understand why farmers might not want to consume crops grown amongst the dead for a variety of reasons, and cannot say I would be different!

That said, I think the paper is really interesting and I want to encourage the authors. I have made a series of notes on the PDF that I hope will help them revise this work. I don't know why the transcripts can not be made available with all identifying information removed, because these transcripts are the actual data to support what the authors are proposing here.

**We have written down the suggestions and corrections from the Review Team in the manuscript. We are very grateful for the help, direction, and correction from the Review Team in helping to refine the manuscript which aims to perfect the manuscript we sent.**

6. PLOS authors have the option to publish the peer review history of their article ([what does this mean?](https://journals.plos.org/plosone/s/editorial-and-peer-review-process#loc-peer-review-history)). If published, this will include your full peer review and any attached files.


If you choose “no”, your identity will remain anonymous but your review may still be made public.


**Do you want your identity to be public for this peer review?** For information about this choice, including consent withdrawal, please see our [Privacy Policy](https://www.plos.org/privacy-policy).

Reviewer #2: No


[NOTE: If reviewer comments were submitted as an attachment file, they will be attached to this email and accessible via the submission site. Please log into your account, locate the manuscript record, and check for the action link "View Attachments". If this link does not appear, there are no attachment files.]

While revising your submission, please upload your figure files to the Preflight Analysis and Conversion Engine (PACE) digital diagnostic tool, <https://pacev2.apexcovantage.com/>. PACE helps ensure that figures meet PLOS requirements. To use PACE, you must first register as a user. Registration is free. Then, login and navigate to the UPLOAD tab, where you will find detailed instructions on how to use the tool. If you encounter any issues or have any questions when using PACE, please email PLOS at [figures@plos.org](mailto:figures@plos.org). Please note that Supporting Information files do not need this step.
