# Supplementary material for: The impact of liquefaction disaster on farming systems at agriculture land based on technical and psychosocial perspectives
Source: PLoS One. 2021 Jan 25;16(1):e0245591. doi: 10.1371/journal.pone.0245591 (PMC7834136; doi:10.1371/journal.pone.0245591)
Supplement: Attachment — Submitted filename: Reviewers.docx [file pone.0245591.s005.docx]

**Reviewers' comments:**

***Reviewer's Responses to Questions***

**Comments to the Author**

1. If the authors have adequately addressed your comments raised in a previous round of review and you feel that this manuscript is now acceptable for publication, you may indicate that here to bypass the “Comments to the Author” section, enter your conflict of interest statement in the “Confidential to Editor” section, and submit your "Accept" recommendation.

**Reviewer #2: (No Response)**

**Reviewer #3: (No Response)**

**To response reviewer:**

**2. Is the manuscript technically sound, and do the data support the conclusions?**

The manuscript must describe a technically sound piece of scientific research with data that supports the conclusions. Experiments must have been conducted rigorously, with appropriate controls, replication, and sample sizes. The conclusions must be drawn appropriately based on the data presented.

**Reviewer #2: Yes**

**Reviewer #3: Partly**

**3. Has the statistical analysis been performed appropriately and rigorously?**

**Reviewer #2: Yes**

**Reviewer #3: Yes**

**4. Have the authors made all data underlying the findings in their manuscript fully available?**

The PLOS Data policy requires authors to make all data underlying the findings described in their manuscript fully available without restriction, with rare exception (please refer to the Data Availability Statement in the manuscript PDF file). The data should be provided as part of the manuscript or its supporting information, or deposited to a public repository. For example, in addition to summary statistics, the data points behind means, medians and variance measures should be available. If there are restrictions on publicly sharing data—e.g. participant privacy or use of data from a third party—those must be specified.

**Reviewer #2: Yes**

**Reviewer #3: Yes**

**5. Is the manuscript presented in an intelligible fashion and written in standard English?**

PLOS ONE does not copyedit accepted manuscripts, so the language in submitted articles must be clear, correct, and unambiguous. Any typographical or grammatical errors should be corrected at revision, so please note any specific errors here.

**Reviewer #2: Yes**

**Reviewer #3: No**

**6. Review Comments to the Author**

Please use the space provided to explain your answers to the questions above. You may also include additional comments for the author, including concerns about dual publication, research ethics, or publication ethics. (Please upload your review as an attachment if it exceeds 20,000 characters)

**Reviewer #2: I suggest to edit few minor language errors**

**Reesponse to reviewer #2:**

**Author :** Thank you for reviewer 2. We have corrected some minor errors in the manuscript by re-reading it carefully and details according this suggestion.

**Reviewer #3**: Sadly, I cannot recommend publication. The paper is plagued with grammatical errors, beginning with the title, and persisting throughout. The research method failed to provide a relevant pre-earthquake baseline with which to evaluate the results - for example, what % of farmers consumed the agricultural products from their lands prior to the earthquakes and thus how have the earthquakes changed this? The authors still adhere to the statement that "This attitude reflects the low-level knowledge of the farmers" and thus the authors' perspective of what constitutes 'knowledge' remain unchanged despite my prior recommendations. The paper floats between an agricultural piece on fertilizer and a social science piece on cultural attitudes but ultimately neither section fulfills its potential nor are clear lines drawn to connect these attributes. The authors should consider submitting this work to a lower tier journal. I really wanted to be able to support publication of this work because it has some interesting components, but ultimately I cannot.

**Response to reviewer #3:**

**Author:** (A) In principle, the method we use has provided steps that can answer problems found in the field, especially related to the comparison or comparison of how many farmers consume their agricultural products before and after the earthquake and liquefaction. Before the disaster 100% of the farmers consumed their agricultural products and after the disaster no one wanted to consume their agricultural products because they believed that the land where they were farming there were still many corpses. Therefore they assumed that the remains of the corpses had been absorbed by plants. So that they are considered to have polluted the land and agricultural products, which is why they are reluctant to consume their agricultural products, especially to provide family needs. Almost all of them sell their agricultural products to traditional markets around the areas affected by the earthquake and liquefaction.

**(B)**  It can be reiterated that all the farmers affected by the earthquake and liquefaction did not complete their education in elementary school. Some even received primary school education but also dropped out or did not graduate, so their insights and knowledge were still very limited. Therefore, even though they are given guidance and counseling, it is difficult to accept it, especially with regard to things that are mystical or superstitious, especially those related to corpses in the ground due to the disaster.

**7. PLOS authors have the option to publish the peer review history of their article (what does this mean?). If published, this will include your full peer review and any attached files.**

If you choose “no”, your identity will remain anonymous but your review may still be made public.

Do you want your identity to be public for this peer review? For information about this choice, including consent withdrawal, please see our Privacy Policy.

**Reviewer #2: No**

**Reviewer #3: No**
